# Recovery of Native Genetic Background in Admixed Populations Using Haplotypes, Phenotypes, and Pedigree Information – Using Cika Cattle as a Case Breed

**DOI:** 10.1371/journal.pone.0123253

**Published:** 2015-04-29

**Authors:** Mojca Simčič, Anamarija Smetko, Johann Sölkner, Doris Seichter, Gregor Gorjanc, Dragomir Kompan, Ivica Medugorac

**Affiliations:** 1 Department of Animal Science, Biotechnical Faculty, University of Ljubljana, Domžale, Slovenia; 2 Division of Livestock Science, Department Sustainable Agricultural Systems, University of Natural Resources and Life Sciences Vienna, Vienna, Austria; 3 Tierzuchtforschung e.V. München, Grub, Germany; 4 Chair of Animal Genetics and Husbandry, Faculty of Veterinary Medicine, Ludwig-Maximilians-University Munich, Munich, Germany; BiK-F Biodiversity and Climate Research Center, GERMANY

## Abstract

The aim of this study was to obtain unbiased estimates of the diversity parameters, the population history, and the degree of admixture in Cika cattle which represents the local admixed breeds at risk of extinction undergoing challenging conservation programs. Genetic analyses were performed on the genome-wide Single Nucleotide Polymorphism (SNP) Illumina Bovine SNP50 array data of 76 Cika animals and 531 animals from 14 reference populations. To obtain unbiased estimates we used short haplotypes spanning four markers instead of single SNPs to avoid an ascertainment bias of the BovineSNP50 array. Genome-wide haplotypes combined with partial pedigree and type trait classification show the potential to improve identification of purebred animals with a low degree of admixture. Phylogenetic analyses demonstrated unique genetic identity of Cika animals. Genetic distance matrix presented by rooted Neighbour-Net suggested long and broad phylogenetic connection between Cika and Pinzgauer. Unsupervised clustering performed by the admixture analysis and two-dimensional presentation of the genetic distances between individuals also suggest Cika is a distinct breed despite being similar in appearance to Pinzgauer. Animals identified as the most purebred could be used as a nucleus for a recovery of the native genetic background in the current admixed population. The results show that local well-adapted strains, which have never been intensively managed and differentiated into specific breeds, exhibit large haplotype diversity. They suggest a conservation and recovery approach that does not rely exclusively on the search for the original native genetic background but rather on the identification and removal of common introgressed haplotypes would be more powerful. Successful implementation of such an approach should be based on combining phenotype, pedigree, and genome-wide haplotype data of the breed of interest and a spectrum of reference breeds which potentially have had direct or indirect historical contribution to the genetic makeup of the breed of interest.

## Introduction

After domestication in the near east cattle became widespread in Europe along the Danubian and Mediterranean route [1]. During the spread across Europe the populations of cattle experienced the harsh Balkan region, the high Alpine region, and finally reached the middle European lowlands where many modern cattle breeds were developed through selection for high production. The industrial revolution and growth of cities demanded higher efficiency of agriculture. Breeders started to increase and consolidate their herds and consequently first herd books were established in regions with growing demand for food [2]. Natural and artificial selection led to the creation of the first breeds from local populations reared close to these regions. On the other hand, sparsely populated areas far from densely populated cities remained behind with scarcely selected heterogeneous local populations of domestic animals. Progressive growth of global human population, improvement of transport and trade initiated improvement of numerous local populations by importing sires of concurrently well-known breeds. The primary loss of genetic diversity within these species is therefore due to progressive upgrading and replacement of local strains with the imported germplasm of highly selected breeds instead of laborious and slow improvement of local populations based on the local agricultural infrastructure (e.g., [3]). In practice, the accelerated achievements of the short-term breeding objectives are directly related to the genetic homogenization of livestock species and the loss of cultural and traditional values of the respective regions [4,5]. The genetic homogenization of livestock is a special form of biotic homogenization which is considered as one of the most prominent forms of the biotic impoverishment worldwide. This process could even endanger the long-term success of species (populations) that are seemingly the ‘winners’ in the homogenization process [6].

In this study, the Cika cattle breed was chosen as an example that embodies the processes outlined above. Cika cattle originated from local single-coloured cattle in the region of today’s Slovenia. To improve milk yields and body frame breeders were upgrading local cows with more productive Mölltaler sires during the second half of the 19^th^ century [7,8]. The Mölltaler breed was assimilated in the Pinzgauer herd-book in 1925 [9] due to the similar red pied sided coat colour pattern [10], although the Mölltaler breed had a lighter body frame and higher milk production. Thereafter, Pinzgauer sires were used for the continuous upgrading of the Cika breed. After the Second World War upgrading with Pinzgauer was discouraged in favour of displacement crossing with the Simmental (Fleckvieh) breed or replacing with the Braunvieh breed depending on the area and breeding policy [7,8]. This change led to near extinction of the Cika breed. In the meantime, the Pinzgauer population was upgraded with the Red Holstein (RHF) breed on a large scale to improve milk production [9]. This contributed to an indirect gene flow from the RHF also into the Cika population. In 1992, a small number of lowland farms held only about 60 Cika cows admixed with Pinzgauer [11]. These animals were registered in the herd book and had therefore known pedigree. In addition, there were about 300 potentially pure Cika cattle in mountain farms preserved by breeders who preferred natural service with Cika sires but kept no pedigrees. In the last two decades Cika, like other local breeds facing extinction, is gaining popularity and some of these populations are increasing in number due to the higher awareness of farmers about the advantages of their traditional breeds (genetic, cultural and economic) and due to the financial incentives to conserve animal genetic resources.

There are a large number of local domesticated populations of various species worldwide that share commonality with the Cika breed in terms of the processes that shaped their current genetic makeup. Some of them were incorporated into large cosmopolitan populations of currently popular breeds [4]. Some populations, however, exist as separate strains or breeds with various degrees of the admixture. In some cases, breeder associations and governments initiated conservation programs to protect and recover such populations. However, there is considerable discussion about the value and effectiveness of such programs and how such programs should be applied in practice [3].

The aim of this study was to obtain unbiased estimates of the diversity parameters, the population history, and the degree of the admixture in Cika cattle using genome-wide marker data. The Cika breed was taken as a representative of local breeds at risk of extinction and with an unknown degree of the admixture. The genetic analyses presented here are of broad interest due to (i) the known local origin, (ii) the well documented admixture process in one part of the population with deep pedigree records, (iii) the unknown admixture in the other part of the population without pedigree records, and (iv) the availability of type trait classification for the entire population. To assess admixture and diversity parameters 14 reference populations were included. These breeds represented possible sources of both admixture and different levels of artificial selection encompassing highly selected single-purpose dairy breeds, moderately selected dual-purpose Alpine breeds, western neighbors predominantly selected for meat traits, scarcely selected Balkan breeds and one African taurine breed as outlier. To obtain unbiased estimates of diversity parameters short haplotypes were used instead of single SNPs to avoid the ascertainment bias of the BovineSNP50 array. Finally, we discuss a strategy for conservation and revitalisation of Cika and equivalent domestic populations taking into account all the available data.

## Materials and Methods

### Ethics statement

**Fig 1:**
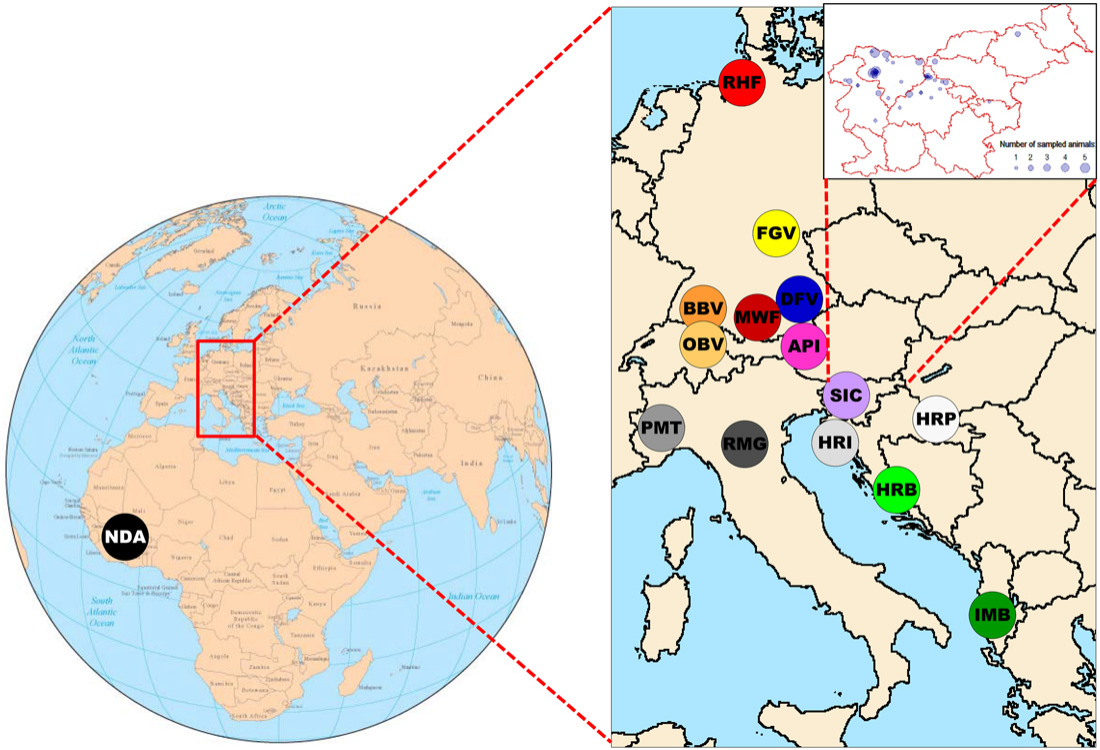
Geographical origin of sampled cattle breeds and more precise sampling locations for Cika within Slovenia. RHF, Red Holstein; FGV, Franken Gelbvieh; DFV, German Fleckvieh; MWF, Murnau—Werdenfelser; BBV, Braunvieh; OBV, Original Braunvieh; API, Pinzgauer; SIC, Cika; HRI, Istrian cattle; HRP, Slavonian—Syrmian Podolic; PMT, Piedmontese; RMG, Romagnola; HRB, Croatian Buša; IMB, Illyrian Mountain Buša; NDA, N’Dama.

For samples used in this study no specific permission or ethical approval was required. As specified below we only used already existing DNA from regularly sampled blood and semen samples that had already been used in previous studies. More precisely, DNA samples from the cattle breeds Red Holstein (RHF), Franken Gelbvieh (FGV), German Fleckvieh (DFV), Murnau—Werdenfelser (MWF), Braunvieh (BBV), Original Braunvieh (OBV) and Illyrian Mountain Buša (IMB) were used in number of published studies [3,12,13,14,15]. DNA samples from three Croatian cattle breeds Istrian cattle (HRI), Slavonian—Syrmian Podolic (HRP), Croatian Buša (HRB) were used in two published studies [3,16]. DNA samples from Pinzgauer (API) were used in study published by Ferenčaković et al. [17] and DNA from the case breed, Slovenian Cika (SIC), was used in microsatellite analyses by Simčič et al. [8]. Piedmontese (PMT), Romagnola (RMG) and N’Dama (NDA) genotypes were kindly provided by R.D. Schnabel (University of Missouri, Columbia, USA) and M. Gautier (INRA, CBGP; France). Collection of the original samples was conducted exclusively during regular quality control of the breeding records. These regular quality controls include paternity and identity testing and are organised by the respective breeding associations. Paternity testing involves blood and semen samples. Blood sampling was conducted by veterinarians using approved procedures that avoid unnecessary pain, suffering and damage and which are in accordance with the German Animal Welfare Act. Semen samples were collected by approved commercial artificial insemination stations as part of their regular reproduction and breeding management in the cattle industry. All samples and data analysed in the present study were obtained with the permission of breeders, breeding organizations and researchers. This study did not involve endangered or protected species. Sampling locations of all samples are shown more precisely in Fig 1.

### Animals and breeds

Cika cattle are the autochthonous cattle breed in Slovenia with a part of population having evident admixture with the Pinzgauer breed. In 2013, the population size of Cika was 3,097 animals in total with 1,750 cows and breeding heifers. Breeding animals (potentially pure and evidently admixed) are divided into three different types based on the available pedigree information and the type traits classification assigned according to body frame size and other morphological traits, as detailed below.

Blood samples from 38 Cika cows and 16 Cika natural service sires were collected from several farms in Slovenia (Fig 1). To obtain representative sample breeding area, number of Cika cattle on the farm and the age of animals were taken into consideration. Additionally, 22 semen samples of Cika sires used for insemination in the population in recent years were obtained from the insemination centre. Based on the known pedigree data a number of sampled animals were related. The majority of animals (46) were considered as purebred (100%) Cika, 6 animals had less than 12.5% of Pinzgauer contribution, while 24 animals had between 14% and 78% of Pinzgauer contribution. All 76 sampled animals had type trait classification and were grouped in the Cika type (59), Semi-Cika type (6), and Pinzgauer type (11).

To integrate the limited information on the case breed into a broader genetic context, the analysis of genetic variability also included 14 breeds covering an area from the Balkans and Italian Peninsula across the Alps to the North Sea. Additionally, taurine cattle breed N’Dama (NDA) from Africa (Burkina Faso, Guinea and Ivory Coast) was included as outgroup necessary for some phylogenetic analyses (Table 1, Fig 1). Two Buša (written also as Busha or Buscha) breeds (IMB from Albania and HRB from Croatia) as well as two Podolian breeds from Croatia (HRI and HRP) were considered as southern Cika neighbours. Two Podolian breeds (PMT and RMG) from Italy were considered as western Cika neighbours. Pinzgauer (API) from Austria was used for upgrading the Cika population in the past. Cika and Pinzgauer (API) share similar pied sided coat colour pattern, while the difference in body frame size and some morphological characteristics are evident. Red Holstein (RHF) is included based on its indirect gene flow into the Cika population. Dual-purpose Original Braunvieh (OBV) and Braunvieh upgraded by Brown Swiss (BBV) were suggested as breeds for replacing Cika while German Fleckvieh (DFV) was suggested for displacement crossing of Cika cattle. Additionally, two German dual-purpose breeds, Franken Gelbvieh (FGV) and Murnau-Werdenfelser (MWF), were included as potential indirect historical contributors. Description of the origin of the breeds and the samples, as well as the breeding purpose and number of samples are shown in Table 1. We used pedigree records (RHF, FGV, DFV, MWF, BBV, OBV, API, and SIC) as well as written and oral evidences from the animal owners (HRI, HRP, HRB, and IMB) to sample mostly purebred and unrelated animals.

**Table 1:** Sample collection: Breed name and code, breeding purpose, sample size (*N*), origin of the breed and samples.

Breed	Code	Breeding purpose	Origin of breed	Published in	*N*
Red Holstein	RHF	Dairy	Germany	[3,12,13,14]	50
Franken Gelbvieh	FGV	Dairy, beef	Germany	[3,12,13,14]	49
German Fleckvieh	DFV	Dairy, beef	Germany	[3,12,13,14]	50
Murnau—Werdenfelser	MWF	Dairy, beef	Germany	[3,12,13,14]	46
Braunvieh	BBV	Dairy	Germany	[3,12,13,14,15]	50
Original Braunvieh	OBV	Dairy, beef	Germany, Switzerland	[3,12,13,14,15]	35
Pinzgauer	API	Dairy, beef	Austria	[17]	50
Cika	SIC	Dairy, beef	Slovenia	[8]	76
Istrian cattle	HRI	Work, beef, (dairy)	Croatia	[3,16]	30
Slavonian—Syrmian Podolic	HRP	Work, beef	Croatia	[3,16]	24
Piedmontese	PMT	Dairy, beef	Italy	[35,55]	22
Romagnola	RMG	Beef	Italy	[35,55]	21
Croatian Buša	HRB	Dairy, beef, (work)	Croatia	[3,16]	24
Illyrian Mountain Buša	IMB	Dairy, beef, work	Albania	[3,12,13,14]	43
N’Dama	NDA	Dairy, work, beef	Burkina Faso, Guinea, Ivory Coast	[35,55]	37

### SNP and haplotype analyses

All 607 animals (Table 1) were genotyped with the Illumina BovineSNP50 BeadChip array using standard procedures (http://www.ilumina.com). Quality control procedures excluded SNPs with genotyping errors (based on available genotypes of relatives), unknown chromosomal position according to the *Bos taurus* genome assembly UMD 3.1 (http://www.cbcb.umd.edu/research/bos_taurus_assembly.shtml#1; autosomal SNPs only), call rate <95%, minor allele frequency <0.025, and the departure from Hardy Weinberg equilibrium within breed (*P*<0.01). Finally, 44,496 autosomal SNPs were considered for the analysis with an average marker density of 56.4 kb.

For haplotype analysis, SNP haplotypes were inferred and missing genotypes were imputed using hidden Markov model of the *Beagle* software package [18]. Three cohorts were formed, consisting of trios (two parents and one offspring), pairs (one parent and one offspring), and unrelated animals, respectively. Included were also those animals that turned out not to be relevant for this study (7,032 animals in total) but had potential to improve haplotyping accuracy [18]. Genome-wide relationships between individuals were estimated using the method of Powell et al. [19] applied to 44,496 SNP genotypes of 607 animals.

Most phylogenetic analyses were based on a smaller subset of 557 animals since they require representative sample of respective population, where a set of 76 Cika animals was reduced to a set of 26 most unrelated purebred animals and likewise for the other breeds. This selection was based on available pedigree information and genome-wide relationship matrix.

### Haplotype diversity

First, the genome was divided into non-overlapping blocks comprised of four SNPs for which the inter-marker distance was less than 50 kb for neighbouring SNPs. We then considered 4,972 blocks as alleles in further analysis. Using short haplotype blocks (maximal length <150 kb) as multi-allelic markers enabled the use of various methods developed for biochemical and microsatellite markers over the past decades.

### Genetic variability

The number of distinct haplotypes across and within each breed for each 4-SNP-block was counted. These counts and derived allele frequencies were used for the estimation of genetic variability parameters. Initially, a set of 99 random blocks was selected for the purpose of testing using *Fstat* v.2.9.3 [20]. Identical results from a standard software packages and our own software ensured reliability of the application used here. In the second phase, our validated programs (suitable for larger datasets) were applied on an entire set of 4,972 blocks. The following statistics were collected: the total number of observed alleles (*nA*), mean number of alleles per block (*mA*), number of private alleles (*npA*, i.e., alleles observed in one subpopulation only), number of common alleles (*ncA*, observed in all subpopulations), and rare alleles (*nrA*, i.e., alleles observed in two subpopulations only). This definition of rare alleles largely implies private alleles introgressed from a donor breed into a recipient breed at a lower frequency by crossing. Since the number of distinct haplotypes is influenced by the sample size, we estimated allelic richness (*AR*) [21] too. Furthermore, estimates of genetic variability, observed (*H*_*O*_) and expected heterozygosity (*H*_*E*_) [22], as well as *F*-statistics [23] for each block including population pairwise *G*_*ST*_ [24,25] were determined. To take into account the concerns about the reliability of classical population differentiation measures like *G*_*ST*_ [24,25], we predicted the true population differentiation *D*_*EST*_ [25] as the harmonic mean of *D* values across loci using the approach described by Crawford [26].

### Cluster analysis

Overall relationships between the breeds were estimated from the *D*_*A*_ distances [27], while the estimation of genetic distances between individuals (*D*_*PS*_ = ln(*PS*)) was based on the proportion of shared alleles, *PS* [28]. A Neighbour network was constructed and plotted with the *SplitsTree4* program [29]. A heuristic approach, as described in Veit-Kensch et al. [30], was used to present the individual *D*_*PS*_-distance matrix in the two-dimensional (2D) space. The great deluge algorithm described by Veit-Kensch et al. [30] uses the first two principal components as a starting configuration and further improves it by maximizing the correlation between the multidimensional distance matrix and Euclidean distance matrix of a 2D-plot. This heuristic algorithm aims to organize individuals into groups without prior knowledge of individuals’ membership to the predefined breeds or groups. For this reason, the resulting 2D-plot could be seen as a product of unsupervised clustering. This analysis was carried out in a complete set of 607 and in a reduced set of 557 animals. The graphical presentations of 2D-plots of *D*_*PS*_-distance matrix were generated using the R programing language [31].

While above genetic distances and clustering methods rely on short haplotypes, two following clustering methods, *Admixture* and *TreeMix*, are based on SNP genotypes. First, unsupervised clustering was performed on the 44,496 autosomal SNPs in both sets of animals (607 and 557) by the *Admixture* program [32] that adopts the likelihood model implemented in the *Structure* program [33,34] but runs considerably faster. To determine the most likely number of clusters (*K*) in our datasets, we estimated cross-validation error based on 20-fold cross-validation. The best value of *K* exhibited the lowest cross-validation error compared to other *K* values. To improve presentation of the *Admixture* results based on a larger set of Cika animals the input data were sorted by breed proportions estimated from pedigree—from those of 100% pure Cika to those containing up to 78% of Pinzgauer genotype, and likewise for Cika crossbreeds with Red Holstein. The *Admixture* results were presented in a stacked barplot generated using the R programming language [31].

Second, inference of population splits and mixtures based on allele counts of the same set of 44,496 autosomal SNPs and using only design with 557 unrelated animals grouped into 15 breeds was performed by the *TreeMix* program [35]. To set position of the root in the maximum likelihood (ML) tree we define N’Dama, a cattle breed from Africa, as the outgroup population. Furthermore, in two alternative analyses we add one or three migration events in the ML tree, respectively. To account for linkage disequilibrium we used blocks of 100 nearby SNPs in all *TreeMix* analyses.

## Results

The average genome-wide relationship coefficient for all 76 Cika animals was -0.013 (± 0.063) with the maximum of 0.68. To create a subset of the most unrelated purebred Cika animals required for phylogenetic analyses we removed highly related animals (relationship coefficient > 0.25), and animals that had exceptionally high relationship coefficient (2 SD above average) compared to the animals from other breeds. Eleven Cika animals were inbred (self-relationship coefficient > 1.05) as well as highly related (> 0.25) to one or more Cika animals. In these pairs the preference was to exclude inbred animals. With the gradual improvement of the pedigree records it became clear that some of these animals are highly admixed. Seventeen Cika animals were misclassified due to the missing or false pedigree data. Among these, 15 were misclassified as pure Cika type but contained 7%-50% API genes and two pure Cika were misclassified as Semi-Cika type. Consequently, at the beginning of the construction of the reduced dataset we excluded 24 admixed animals based on the known pedigree, which contained more than 12.5% of foreign genes. This exclusion was supported by the estimated genome-wide relationships. Furthermore, the analysis of the genome-wide relationship matrix, *D*_*PS*_-distance matrix in the two-dimensional space, and the *Admixture* results between all the remaining animals indicated seven additional Cika animals as related to one or more Alpine breeds (DFV, FGV, OBV and MWF). Even though the background of these relationships in most cases remains unknown we also excluded these animals from the phylogeny analysis and suggest excluding these from the conservation nucleus as well. Combination of all these exclusions led to the final sample of 26 most unrelated purebred Cika animals. The genome-wide relationship coefficient among these 26 Cika animals was on average very low -0.039 (± 0.073) with the maximum of 0.25. This set included only seven genome-wide relationship coefficients over 0.20 (2.2%). Most phylogenetic analyses were based on the reduced subset of 557 animals, i.e., 26 the most unrelated purebred Cika and the similar selection of 531 animals from other 14 comparison breeds. On the other hand, all 76 purebred and admixed Cika animals were included in the larger dataset of 607 animals, which was used for the 2D presentation and the *Admixture* analyses. Throughout the remainder of the text, when dataset is not explicitly mentioned the smaller set is implied.

### Genetic diversity

The number of the common alleles (*ncA*) was 11,231 with 2.26 per haplotype block on average. The *nA*, *mA*, *npA*, *H*_*O*_, *H*_*O[SNP]*_, *H*_*E*_, and *AR* of Cika in comparison with the other 14 breeds are shown in Table 2. The total number of observed alleles (*nA*) and the mean number of alleles (*mA*) per haplotype block ranged from 26,053 and 5.24 in HRP to 44,416 and 8.93 in IMB, respectively, while Cika (*nA* = 34,807; *mA* = 7.00) was in the middle of these extremes. Cika also showed lower *nA* and *mA* compared to API (*nA* = 37,987; *mA* = 7.64). IMB showed the highest number of private (*npA* = 1,307) and rare alleles (*nrA* = 1,467) while BBV had the lowest values (*npA* = 158, *nrA* = 251). The discrepancy between these two extremes is enormous, i.e., the number of private and rare alleles in the highly selected BBV breed is 8.27 and 5.84 times lower, respectively. The second Buša breed (HRB) showed lower but still very high *npA* (875) and *nrA* (801). However, Cika showed the largest difference between *npA* (237) and *nrA* (422). Among all 15 breeds only in two Bušas (HRB and IMB) and N’Dama were *H*_*O*_ lower than *H*_*E*_. Only IMB (0.743) and PMT (0.738) showed higher *H*_*O*_ compared to Cika (0.737). Allelic richness, a diversity measure not influenced by sample size, was the highest in HRB (7.771) and the lowest in HRP (5.101). Cika had allelic richness (6.695) similar to API (6.492), HRI (6.735) and PMT (7.141), which are the three geographically closest breeds. Allelic richness showed highest correlation with *mA* (0.90) and *H*_*E*_ (0.88) but lowest with *H*_*O[SNP]*_ (0.53).

**Table 2:** Summary statistics—neutral genetic diversity of 15 cattle breeds.

Breed	*N*	*nA*	*mA*	*npA*	*nrA*	*H*_*O*_	*H*_*O[SNP]*_	*H*_*E*_	*AR*
RHF	50	34474	6.93	465	602	0.709	**0.342**	0.703	6.050
FGV	49	35178	7.08	319	472	0.713	0.334	0.699	6.160
DFV	50	34674	6.97	238	393	0.700	0.326	0.687	6.018
MWF	46	31627	6.36	227	306	0.717	0.334	0.681	5.594
BBV	50	31006	6.24	158	251	0.660	0.307	0.647	5.355
OBV	35	33344	6.71	251	367	0.701	0.325	0.688	6.082
API	50	37987	7.64	451	676	0.716	0.335	0.706	6.493
SIC	26	34807	7.00	237	422	**0.737**	**0.343**	0.718	6.695
HRI	30	36364	7.31	380	603	0.719	0.331	0.711	6.735
HRP	24	26053	5.24	219	299	0.681	0.312	0.642	5.101
PMT	22	36084	7.26	430	526	0.738	0.343	0.721	7.141
RMG	21	30170	6.07	305	368	0.680	0.312	0.667	6.031
HRB	24	**40116**	**8.07**	**664**	**847**	0.702	0.324	**0.746**	**7.771**
IMB	43	**44416**	**8.93**	**1307**	**1467**	**0.743**	**0.344**	**0.748**	**7.620**
NDA		**30135**	**6.06**	**875**	**801**	**0.581**	**0.237**	**0.589**	**5.332**
All	477	58632	11.79	6526	4200	0.699	0.323	0.690	6.279

The sample size (*N*), total number of observed alleles (*nA*), mean number of alleles per haplotype block (*mA* = *nA*/ 4817), number of private alleles (*npA*), number of rare alleles defined as alleles observed in only two subpopulations (*nrA*), average observed heterozygosity (*H*_*O*_), *H*_*O*_ estimated from SNP genotypes (*H*_*O[SNP]*_), average expected heterozygosity (*H*_*E*_) and allelic richness (*AR*[21]). RHF, Red Holstein; FGV, Franken Gelbvieh; DFV, German Fleckvieh; MWF, Murnau—Werdenfelser; BBV, Braunvieh; OBV, Original Braunvieh; API, Pinzgauer; SIC, Cika; HRI, Istrian cattle; HRP, Slavonian—Syrmian Podolic; PMT, Piedmontese; RMG—Romagnola; HRB, Croatian Buša; IMB, Illyrian Mountain Buša; NDA, N’Dama.

### Clustering analyses

Pairwise population differentiations are shown as *D*_*EST*_ and *G*_*ST*_ values in Table 3. The correlation between *D*_*EST*_ and *G*_*ST*_ was very high (0.990) but *D*_*EST*_ showed better diversification (from 0.008 to 0.318). The highest average *D*_*EST*_ values were observed among NDA from Africa, autochthonous HRP and highly selected RHF as well as BBV when compared with other breeds (0.174–0.263). The lowest average genetic differentiations were observed for Bušas, PMT and Cika when compared with other breeds (0.076–0.097). Pairwise *D*_*EST*_ value between Cika and API (0.046) was similar to the one between Cika and IMB (0.045) as well as between API and HRB (0.055). The lowest *D*_*EST*_ value when comparing Cika with other breeds was between Cika and HRB (0.022) followed by Cika and PMT (0.033) differentiation. The lowest *D*_*EST*_ when comparing all breeds was between HRB and IMB (0.008), which demonstrates the low genetic differentiation of Buša.

**Table 3:** Pair-wise population differentiations.

	RHF	FGV	DFV	MWF	BBV	OBV	API	SIC	HRI	HRP	PMT	RMG	HRB	IMB	NDA	${\bar{D}}_{EST}$
**RHF**		0.166	0.185	0.190	0.220	0.174	0.153	0.134	0.162	0.241	0.120	0.199	0.103	0.119	0.318	0.177
**FGV**	0.087		0.077	0.129	0.161	0.109	0.112	0.074	0.117	0.207	0.065	0.156	0.061	0.087	0.273	0.128
**DFV**	0.097	0.051		0.125	0.157	0.107	0.119	0.061	0.123	0.213	0.070	0.166	0.065	0.100	0.278	0.132
**MWF**	0.100	0.074	0.075		0.167	0.122	0.134	0.104	0.137	0.223	0.092	0.177	0.084	0.111	0.282	0.148
**BBV**	0.122	0.098	0.099	0.104		0.114	0.167	0.135	0.163	0.253	0.124	0.200	0.121	0.147	0.312	0.174
**OBV**	0.093	0.066	0.067	0.073	0.078		0.115	0.081	0.109	0.205	0.068	0.153	0.058	0.086	0.267	0.126
**API**	0.080	0.065	0.069	0.075	0.099	0.069		0.046	0.109	0.199	0.065	0.150	0.055	0.076	0.266	0.126
**SIC**	0.071	0.047	0.043	0.061	0.082	0.052	0.033		0.078	0.174	0.033	0.122	0.022	0.045	0.243	0.097
**HRI**	0.082	0.065	0.069	0.076	0.095	0.064	0.062	0.047		0.166	0.061	0.102	0.043	0.061	0.240	0.119
**HRP**	0.131	0.118	0.124	0.128	0.152	0.120	0.113	0.099	0.096		0.160	0.210	0.137	0.153	0.293	0.202
**PMT**	0.065	0.043	0.047	0.056	0.077	0.047	0.042	0.028	0.041	0.093		0.099	0.012	0.024	0.227	0.087
**RMG**	0.108	0.092	0.099	0.103	0.124	0.094	0.088	0.074	0.065	0.126	0.065		0.086	0.102	0.256	0.155
**HRB**	0.055	0.037	0.041	0.048	0.070	0.039	0.035	0.019	0.029	0.077	0.015	0.054		0.008	0.204	0.076
**IMB**	0.060	0.048	0.055	0.059	0.081	0.050	0.044	0.030	0.036	0.084	0.022	0.060	0.010		0.219	0.095
**NDA**	0.179	0.163	0.170	0.172	0.197	0.165	0.158	0.145	0.145	0.190	0.138	0.167	0.120	0.125		0.263
${\bar{G}}_{ST}$	0.095	0.075	0.079	0.086	0.105	0.077	0.074	0.059	0.069	0.118	0.056	0.094	0.046	0.054	0.160	

*D*_*EST*_ values as an estimator of true differentiation [24] among 15 breeds are presented above the diagonal and pair-wise *G*_*ST*_ below the diagonal. Average values (${\bar{D}}_{EST}$ and ${\bar{G}}_{ST}$) are presented in the last column and row. RHF, Red Holstein; FGV, Franken Gelbvieh; DFV, German Fleckvieh; MWF, Murnau—Werdenfelser; BBV, Braunvieh; OBV, Original Braunvieh; API, Pinzgauer; SIC, Cika; HRI, Istrian cattle; HRP, Slavonian—Syrmian Podolic; PMT, Piedmontese; RMG, Romagnola; HRB, Croatian Buša; IMB, Illyrian Mountain Buša; NDA, N’Dama

**Fig 2:**
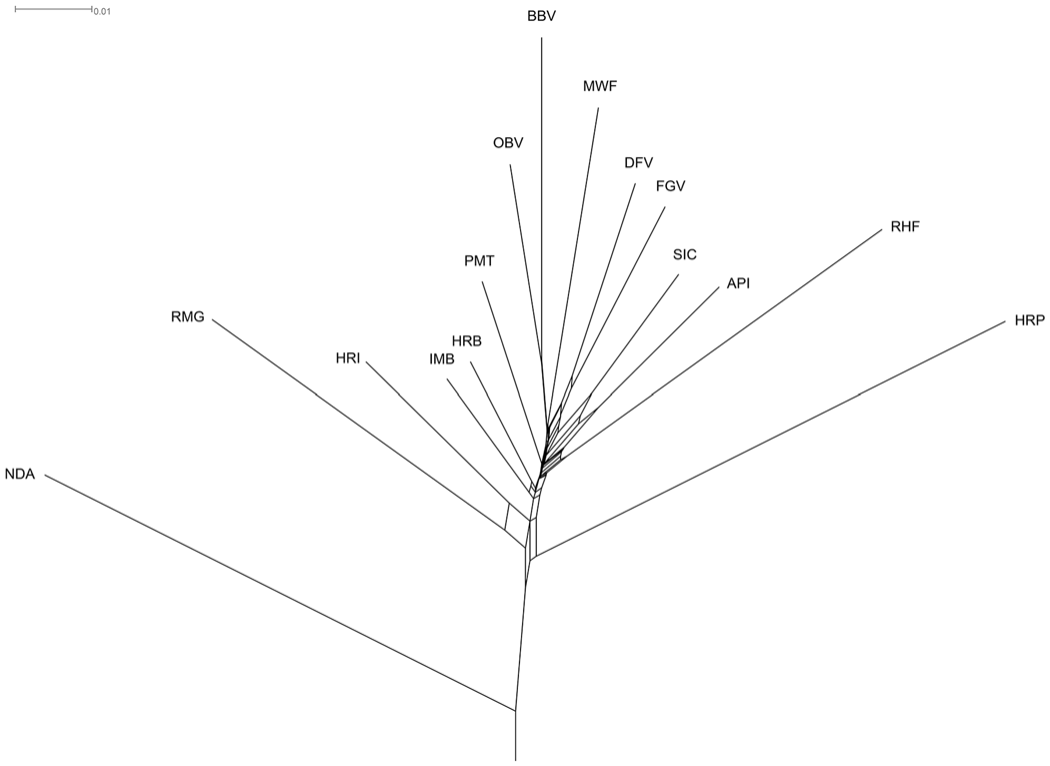
Presentation of Nei’s *DA*-distance matrix as a rooted Neighbour-Net graph. RHF, Red Holstein; FGV, Franken Gelbvieh; DFV, German Fleckvieh; MWF, Murnau—Werdenfelser; BBV, Braunvieh; OBV, Original Braunvieh; API, Pinzgauer; SIC, Cika; HRI, Istrian cattle; HRP, Slavonian—Syrmian Podolic; PMT, Piedmontese; RMG, Romagnola; HRB, Croatian Buša; IMB, Illyrian Mountain Buša; NDA, N’Dama.

Nei’s *D*_*A*_-distances were plotted as a Neighbour-Net (Fig 2) rooted by NDA population. The taurine outgroup from West Africa joined the network of the European cattle breeds at group of three Podolian breeds (HRI, HRP, RMG). The fourth Podolian breed PMT with relatively high allelic diversity (*AR* = 7.141) doesn’t show clear clustering in this design and has a higher genetic similarity to Alpine breeds. The Neighbour-Net graph suggested relatively long-term and close co-evolution of both Braunvieh populations (OBV and BBV). Similarly close but shorter co-evolution is suggested for DFV and FGV. On the contrary, Cika and Pinzgauer showed relatively long but wide phylogenetic connection (Fig 2). Both branches were similarly long. The Cika-Pinzgauer cluster was flanked by RHF with its relatively long branch on the one hand, and by the FGV-DFV cluster on the other. Both Buša breeds, HRB and IMB, were easy to recognise due to their short branches. They were clustered together, but split from each other just after the tree trunk. The longest branches belonged to the HRP and NDA.

Additional to the above mentioned phylogenetic analysis, we inferred patterns of splitting and possible historical mixing between investigated populations with maximum likelihood (ML) methods implemented in the *TreeMix* program. The ML tree, rooted by N’Dama population (S1 Fig), shows highly similar characteristics to the above described Neighbour-Net. Assuming one migration event, we detect relatively recent migration of DFV genes into the Cika breed while remaining structure of tree stays unchanged (S1 Fig). This result is consistent over series of five independent *TreeMix* runs. Assuming two and three migration events leads to inconsistent results over five independent *TreeMix* runs (S1 Fig). Most common feature of these inconsistent runs is suggested migration of early N’Dama-ancestors to the root of the South-East European cattle populations (IMB, HRB and HRP) or to HRB only. Next common feature is suggested migration of PMT ancestors to the root of the HRI-RMG cluster (S1 Fig).

**Fig 3:**
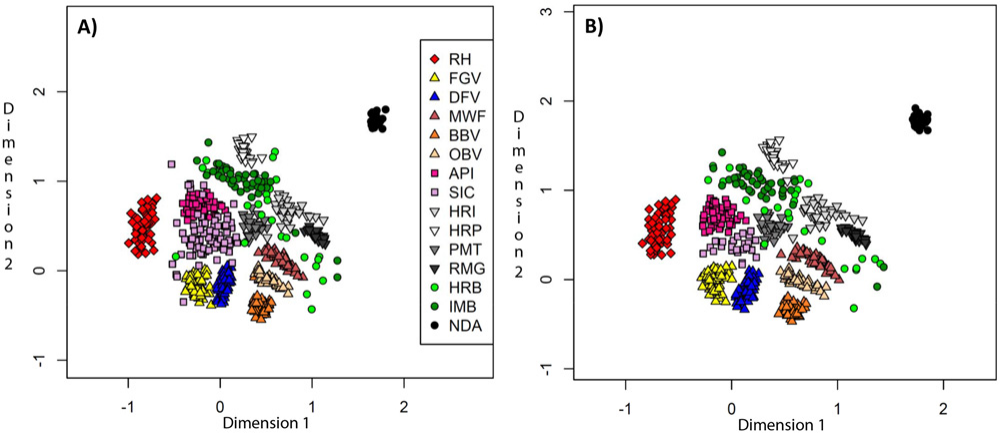
Individual *D_PS_*-distance matrix in the two-dimensional space. A dataset with all 76 Cika animals **(a)** and a reduced dataset with 26 animals **(b)**. RHF, Red Holstein; FGV, Franken Gelbvieh; DFV, German Fleckvieh; MWF, Murnau—Werdenfelser; BBV, Braunvieh; OBV, Original Braunvieh; API, Pinzgauer; SIC, Cika; HRI, Istrian cattle; HRP, Slavonian—Syrmian Podolic; PMT, Piedmontese; RMG, Romagnola; HRB, Croatian Buša; IMB, Illyrian Mountain Buša; NDA, N’Dama.

Fig 3 shows the *D*_*PS*_ distances in the two dimensional presentation (2D). The distribution of the larger set with 607 individuals of 15 breeds is shown in Fig 3a, where Cika population was represented by 76 pure and admixed individuals. Thirteen breeds formed well-recognized clusters and were clearly separated from each other, while Cika and HRB animals were scattered to a large extent. Most HRB individuals were scattered close to the IMB and HRI clusters, while others seemed to be outliers. Cika clustered in the neighbourhood of API with few individuals falling into the API cluster. Some Cika individuals also seemed to be outliers. Fig 3b shows the distribution of the reduced set, where Cika is represented by 26 purebred individuals that were less inbred and were nominally unrelated. Analysis of this data led to the formation of 14 distinct clusters. Pure Cika individuals were clustered independently from the API cluster though in the neighbourhood of the API cluster. Only HRB showed a lot of outliers just like in the Fig 3a and did not form its own cluster.

**Fig 4:**
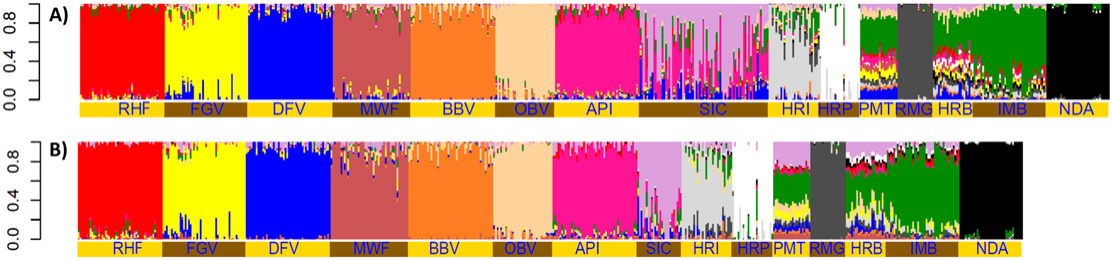
The *Admixture* analyses of two data sets. A dataset with all 76 Cika animals **(a)** and a reduced dataset with 26 animals **(b)**. RHF, Red Holstein; FGV, Franken Gelbvieh; DFV, German Fleckvieh; MWF, Murnau—Werdenfelser; BBV, Braunvieh; OBV, Original Braunvieh; API, Pinzgauer; SIC, Cika; HRI, Istrian cattle; HRP, Slavonian—Syrmian Podolic; PMT, Piedmontese; RMG, Romagnola; HRB, Croatian Buša; IMB, Illyrian Mountain Buša; NDA, N’Dama.

The results of the *Admixture* analysis of the reduced set detected the lowest cross-validation error at *K* = 13. Fig 4 and S2 Fig presents the *Admixture* results for this *K* value in the larger set with 76 Cika animals (Fig 4a) and in the reduced set with 26 Cika animals (Fig 4b). Fourteen breeds created their own clusters and these results suggested that HRB is a synthetic population (Fig 4). In Fig 4a admixture is clearly seen with the API (26%), DFV (9%), Brown breeds (3%), and RHF (2%). Contribution from the Cika’s southern neighbours (HRI-IMB 8%) was also indicated. However, when we considered the reduced set (Fig 4b) the admixture proportions in Cika animals were evidently lower though still present (API 9%, DFV 6%, OBV-BBV 2% and HRI-IMB 5%).

### Correlations between genetic, pedigree and phenotypic parameters

The correlation between type trait classification and the pedigree contributions from Pinzgauer was 0.71 (S1 Table). Likewise, the correlation between type trait classification and the average genome-wide relationship between each Cika animal to all sampled Pinzgauer animals was 0.65, while the correlation between the pedigree contributions from Pinzgauer and average genome-wide relationship coefficient between each Cika animal to all included Pinzgauer animals was nominally higher (0.69). The correlation between type trait classification and the proportion of admixed Pinzgauer genes within Cika individuals from the *Admixture* analysis was 0.64, while the correlation between type trait classification and the proportion of Cika genes from the *Admixture* analysis was 0.53.

S3 Fig shows the *D*_*PS*_ distances in the two dimensional presentation (2D). The dataset included Cika and four breeds (RHF, FGV, DFV and API) with possible direct contribution to the Cika gene pool as identified by genome-wide IBS and IBD matrix. In the three parts of the S3 Fig we highlighted animals selected as pure Cika based on a) combined haplotype, pedigree data and type trait classification, b) pedigree information alone and c) type trait classification alone. The comparison of these figures reveals the subtle differences between separate and combined approach for selection of conservation-worthy animals. Type traits and pedigree information used separately identify Cika animals with API contribution correctly. Only one of 14 animals with significant API contribution was misallocated to pure Cika by type traits classification (S3 Fig). On the other hand, both pedigree information and type traits wrongly placed seven animals with significant contribution of foreign breeds other than API (i.e. DFV, FGV or RHF). Furthermore, combined approach as well as type traits alone classified 13 Cika animals as worth conserving even if the written herd-book records suggested some contribution of API breed. Likewise, two additional animals were classified as pure Cika in spite of type trait classification as Semi-Cika. In total, 54 (71%) of 76 investigated animals were classified as pure Cika by combined approach. Only two third of these (37) would be allocated to pure Cikas by herd-book records or type traits alone.

## Discussion

Cika cattle, like many other local breeds, was under pressure with the introduction of cosmopolitan breeds and was near extinction due to the continuous upgrading with Pinzgauer as well as crossing with Fleckvieh and replacement with Braunvieh. When Cika population was included in the conservation program, doubts have been raised about the uniqueness of the breed and the level of the admixture. The Cika herd book had records on mostly admixed animals with known pedigree going back a number of generations and with relatively accurate estimated proportions of foreign genes from Pinzgauer and Red Holstein. In 2002, potentially pure Cika animals from the mountain area were included in the program as 100% pure Cika based only on type traits classification even though their pedigree was unknown. However, this increased population size but also doubts in the breeding community about the uniqueness of the population.

These conditions partly resemble a curious situation that arose from the restoration and conservation of the Austrian Murbodner (AMB) cattle breed [12]. Original Murbodner cattle were gradually upgraded by Franken Gelbvieh and/or replaced with Fleckvieh after the Second World War. At the beginning of 1980s, some Austrian organisations started to search for the typical AMB animals preferably without the Gelbvieh genes. Since the most typical AMB candidates with extensive pedigrees had one or more Gelbvieh ancestors the selected putative purebred AMB animals predominantly did not have pedigree records. Because both AMB and Gelbvieh show morphological similarity, it was not possible to effectively exclude Gelbvieh admixture in these animals based on the phenotype alone. Recently, phylogenetic results showed large admixture of Gelbvieh genes in the restored AMB population [12]. Despite the current conservation program of AMB as a putative autochthonous cattle breed, in fact it appears nowadays as a subpopulation of Gelbvieh that is also endangered. This demonstrates that upgrading and subsequent selection on the introgressed genes could result in the loss of genetic identity of a specific breed, even if considerable efforts were made to find the original purebred animals. One of the goals of this study was to capitalize on the recent advent of high-throughput genotyping techniques to check if a comparable scenario is probable for Cika and Pinzgauer. With the power of high-throughput data providing accurate genetic structure of the subpopulations [36] and knowledge of actual individual relationships derived from more than 40,000 SNP markers [37], it should be ensured that an appropriate conservation program is in place for Cika.

In order to achieve these conservation goals and to avoid the ascertainment bias of the BovineSNP50 array we used the results based on the 4-SNP-block haplotypes. The BovineSNP50 array was developed mainly on the SNP allele frequency spectrum of highly selected, commercial, and cosmopolitan breeds [38]. Estimates of genetic diversity can therefore be influenced by the ascertainment bias of the array and can even lead to incorrect conclusions [36, 38–41]. On the other hand, haplotype diversity is robust to the marker ascertainment bias and provides more information regarding diversity and population history as shown through simulations and empirical data [42–44]. In spite of this, most currently published studies on cattle diversity are based on the BovineSNP50 genotype data instead of the haplotype data. One exception is the phylogenomic study of ruminants by Decker et al. [41]. Likewise, Amador et al. [45] used haplotypes of 10 consecutive SNPs from the OvineSNP50 BeadChip. The comparisons of diversity parameters or phylogeny based on the SNP allele frequency and the short haplotypes were of secondary interest of this study, but we would like to point out to the difference in the observed heterozygosity (Table 2). Based on haplotypes the highest *H*_*O*_ was obtained for IMB (0.743), while *H*_*O*_ estimated from the SNP genotypes (*H*_*O[SNP]*_) was comparably high in RHF, SIC, PMT and IMB (0.342–0.344). The high *H*_*O[SNP]*_ estimated in RHF sample is in conflict with a relatively low haplotype diversity and could be due to the ascertainment bias. Supporting this result is also the low correlation (0.53) between haplotype allelic richness and *H*_*O[SNP]*_ (Table 2). In order to avoid the ascertainment bias, only results based on the 4-SNP-block haplotypes are discussed below.

Considering the situation that arose when restoring the AMB, the main idea was to combine all of the available data to identify admixed animals which were recorded as purebred Cika based only on type traits classification after their discovery in 2002. In addition, animals with known pedigree can be misclassified due to the errors in the pedigree or the discrepancy between the expected and the actual admixture. With the exclusion of the admixed and highly related Cika animals we wanted to show the phylogenetic characteristic of the original (pure) Cika only as well as to build a knowledge base for later practical conservation work on similar populations. Ability of the methods implemented in the *Admixture* and comparable programs to allocate foreign genome and haplotypes to their respective breed depends on the study design. Our experience suggests that in cases where pedigree is not known, a feasible way to find unknown genetic admixture is to add all possible direct and indirect source populations to the initial dataset. Similar to other endangered breeds with comparable history there is no perfect design and it is likely that the dataset used in this study does not include all breeds from which genes could have been admixed in Cika, e.g. the already extinct Mölltaler breed.

Cika and HRB animals, which were identified as highly inbred by the genome-wide IBD matrix, were outliers in the 2D space, far away from their breed clusters (Fig 3a).These outliers are present in the population of Cika and HRB due to the population fragmentation [16]. Breeders in the marginal mountain areas that prefer local breeds used their own natural service bulls. These bulls were usually related to the dams, which led to inbreeding. This could be a common situation in endangered indigenous breeds under low management level. Cika animals identified as highly inbred were also detected as highly related to one or more other animals in our design. In such a situation we preferred to exclude inbred animal from the set for phylogenetic analyses but not from a conservation program.

According to the expected and observed haplotype heterozygosity, Balkan breeds were more diverse than Alpine breeds and dual-purpose breeds were more diverse than highly selected single-purpose breeds. Large current and past effective population size as well as virtual absence of systematic artificial selection combined with proximity to major centres of domestication in the Fertile Crescent could be main reasons for higher diversity in Buša strains [12,14]. Even if there are no historical records for ancient or recent introgression of *indicine* or African *taurine* ancestry into Balkan cattle breeds (Buša or Podolic) this possibility exists. Furthermore, Balkan cattle population is located closer to current Anatolian cattle that carried both *indicine* and African *taurine* ancestry [46] which could indirectly shape the distribution of rare and private alleles in cattle population of Balkan. If so, we would expect systematic effect in all indigenous Balkan cattle breeds, i.e. increased allelic diversity in both Buša and Podolic cattle. On the contrary, two Balkan breeds (IMB and HRP) are at the opposite ends for allelic diversity according to our results. However, some results of the *TreeMix* procedure support results of [46] and suggest possible migration of earlier N’Dama-ancestors to the root of the South-East European cattle populations (IMB, HRB and HRP). This migration edge added basal to phylogeny, i.e. close to root, indicates that possible admixture occurred very early in time or even from a more diverged population ancestral to both South-East European and African taurine cattle. Be that as it may, the main objective of this study is to verify known admixture in the case breed Cika which was confirmed by *TreeMix* analyses. In addition to the close relationship between Cika and API, *TreeMix* suggests relatively recent mixture with DFV. This could explain higher genetic diversity of current Cika population. On average, the neutral diversity estimators put Cika in the position between the Balkan breeds (Buša and Podolic) and the Alpine breeds, which corresponds with the geographical location of Slovenia as well as with admixed status of Cika.

The high average *D*_*EST*_ values observed between RHF and other breeds (${\bar{D}}_{EST}$ = 0.177; Table 3) may reflect artificial selection, while the high ${\bar{D}}_{EST}$ value for HRP (${\bar{D}}_{EST}$ = 0.202) could reflect long-term isolation of the breed and a limited number of founders after the bottleneck [16]. Cika showed the lowest pairwise *D*_*EST*_ values with HRB (0.022) and PMT (0.033) despite remarkably different breed characteristics (e.g., in horns, coat, and mucosa colour). Furthermore, *D*_*EST*_ value to Buša strain from Albania (0.045) is also lower then to Austrian Pinzgauer (0.046) despite relatively long geographical distance. These low *D*_*EST*_ values to both Buša strains and PMT suggest either low genetic differentiation of all four subpopulations or ancient genetic relationships through past trades which is possible given the geographical proximity. The lowest ${\bar{D}}_{EST}$ values for HRB (0.076), PMT (0.087), IMB (0.095) and SIC (0.097), the broad allelic diversity in all four breeds (Table 2) and the clustering by *Admixture* suggest low differentiation as being more relevant. Despite the verifiable evidence of the direct introgression from Pinzgauer to Cika, it should be considered that the *D*_*EST*_ matrices were prepared with only potentially pure Cika animals. These animals show relatively low genetic differentiation to Pinzgauer (*D*_*EST*_ = 0.046). The key question is which part of relatively high genetic diversity in Cika is caused by admixture and which part represents original neutral diversity of the population under relatively low artificial selection.

Allelic diversity represented by *nA*, *mA*, *npA*, *nrA*, and *AR* (Table 2) clearly suggests two artificially unselected Buša strains as the populations with the highest neutral diversity. On the other hand, one Podolic breed (HRP, weak artificial selection but with known recent population bottleneck, [16]) and one highly selected Alpine breed (BBV, with small effective population size [14]) demonstrated the lowest allelic diversity. Cika shows a low number of private alleles but relatively high *H*_*O*_ and *AR*. Comparing *nrA* and *npA* Cika show highest proportion of rare alleles, defined as alleles observed in only two subpopulations (*nrA/npA* = 1.78). This is due to sharing of 24% rare allele with API, i.e., otherwise private alleles of API or SIC were classified as rare due to sharing. Taken together, these patterns suggest that even the sample of 26 most pure Cika is still partly admixed. Then again, unsupervised clustering performed by *Admixture* and 2D presentation of *D*_*PS*_-distances identified Cika as a breed worth preserving. Even though there are recognisable proportions of Alpine (mainly API and DFV) and southern (IMB, HRI) contributions in most Cika animals, both unsupervised approaches identified Cika as a population with a unique genetic identity. Comparison of the clustering based on the initial set of 76 Cika animals (Fig 3a and Fig 4a) with the results based on 26 selected Cika animals clearly suggest that the multistage procedure used here is able to detect individuals which are more important to preserve. It is important to keep in mind that additional animals, considered as less admixed, were not included in the set of 26 Cika animals due to their high relationship to one or more animals already included in the set. All these currently living, less admixed animals should be considered as Cika nucleus for breeding of sires and bull dams of the next generations and thus recovering the native genetic background of Slovenian Cika. Different de-introgression strategies were developed based on the pedigree information [47], molecular information [45,48,49], or other traits, like breed specific phenotypes as well as productive traits [50]. A combination and improvement of these approaches should be considered for de-introgression of admixed endangered domestic populations. The S1 Fig illustrates advantages of combined approach to the separate usage of pedigree or phenotype information. Practical conservation breeding should inevitably combine all available information but be aware of the ascertainment bias of the most SNP arrays developed for the livestock species. This is especially important if the breed of interest was not involved in the development of the SNP array. Previously published simulation studies [48,49] did not consider this bias. Local breeds like Cika were not considered when Bovine SNP array was designed [38] and consequently their private alleles are not known and not included onto array causing the markers to be on average more informative for the considered popular commercial breeds. On the other hand, Cika and other endangered breeds are facing extinction due to the introgression and replacement by the commercial breeds that contribute their most informative markers onto SNP arrays. Therefore, de-introgression strategies using information on private alleles [48] is currently only of theoretical interest or useful only for the breeds included in the creation of a SNP array, which are mostly not at risk of extinction. Also, the use of breed specific phenotypes (i.e. private phenotypes) for de-introgression [50] will result, at most, in the recovery of certain regions in genome linked to them. For example, there is a large discrepancy between phenotypic and background genetic differentiation of geographically close cattle breeds Blanc-Bleu Belge and Holstein [12,14]. As mentioned before, Austrian Murbodner breeders used a combination of phenotypes and pedigree records to recover the breed, but according to the molecular genetic results the success of recovery following this approach was limited [12]. Our case breed is also extensively phenotyped and breeders are more familiar with using phenotype than haplotype data. However, phenotype information alone is not conclusive though it is important to preserve some essential breed characteristics. Our experiences (S1 Fig) suggest combined use of all the available information with an emphasis on molecular information. In summary, it can be concluded that pedigree data as well as type traits classification identified quite well the animals admixed with the main breed (here API) used for upgrading. Animals with significant proportion of gene introgressed by historical or unexpected crossing (e.g. OBV, BBV, DFV, FGV, RHF) were not identified by pedigree data and/or type traits. Erroneous or incomplete pedigree records lead to misclassification in both directions. For illustration, in this study ten Cika animals were scored as admixed by genealogical coancestry despite being pure according to molecular coancestry. Detailed investigation of affected cases suggests wrong pedigree arising from the use of Cika bull for natural mating after official insemination by API sire was not successful. Additionally, we recognized the risk of misclassification of some purebred animals kept in well controlled environment as Semi-Cika. This is due to the subjective expectation of the assessor that pure indigenous animal should be smaller sized or with lower production then observed. These biased expectations present a common threat at phenotypic evaluation of endangered, indigenous, domestic populations. Making such mistakes leads to double losses, i.e. we lose preferential indigenous variants able to compete with common commercial breeds.

Combined use of all the available information with an emphasis on molecular information should bear on haplotypes, short or long, depending on the characteristics of introgression events. The successful identification and the removal of exogenous haplotypes present in an admixed population will depend on the availability of high quality haplotypes of case and source populations. In this case study we detected admixed and not related animals with large exogenous haplotypes at different chromosomes (data not shown). Appropriate inclusion of progenies from these individuals (via regular or target mating) into the nucleus could effectively remove exogenous haplotypes and avoid increase in inbreeding. Likewise missing and wrong information in the incomplete pedigree records could be efficiently improved and corrected based on the molecular composition of a breed. Generally, the advantage of using molecular information increased with the increase in SNP density. In scenarios evaluated by [51] the SNP density should reach at least 3 times the effective population size per Morgan or about 500 SNPs/Morgan. As repeatedly confirmed larger reference panels and high marker density substantially increase haplotype quality and imputation accuracy, particularly for low-frequency variants [18]. Therefore, unconsolidated indigenous breeds with large population size whose private or rare alleles were not involved in the development of the SNP array need even higher SNP density to permit high quality haplotyping. Many livestock breeds under the pressure or even under the threat of extinction are indigenous local strains structured in more diffuse subpopulations, like Pramenka sheep [52] or Buša cattle [3]. Such populations are characterised by a low breeding level in the past, resulting in high neutral diversity and a large effective population size [3]. On the contrary, exogenous haplotypes come from relatively compact and administratively isolated commercial populations. Therefore, the de-introgression of these loosely defined and before admixture highly diverse populations should rely less on the search for the original genetic background of a native population but rather on the identification and removal of haplotypes from exogenous compact common breeds. Our case population shows most of these characteristics and is a suitable candidate for the implementation of the conservation and recovery approach, combining all the available phenotype, pedigree, and high-density haplotype information. Successful de-introgression process will, however, be influenced by the differentiation among the target population and populations they have admixed with. The higher the similarity, the more difficult it is to differentiate between both populations [45] and more dense information (molecular, genealogical and phenotypical) is necessary. As demonstrated by analyses of composite Swiss Fleckvieh breed [53], when pedigree records of admixed animals were deep and known to their pure ancestors then the correlation between pedigree-based and genome-based admixture was very high (0.972). However, the common characteristic of the endangered indigenous breeds under low management level is absence of such dense genealogical information. Also, the dataset of 76 Cika with relatively good quality of pedigree records contained 13 animals with unknown both parents as well as six animals with unknown sire. All these were recorded as 100% pure Cika in a herd book. Consequently, the correlation between pedigree-based and genome-based admixture of 76 Cika animals was lower (0.72). It needs to be repeatedly emphasized that studies estimating parameters and developing tools (e.g. [53] by necessity use designs with denser information and by the majority include samples of cosmopolitan breeds where lower effect of ascertainment bias could be expected then in the indigenous breeds.

Populations of livestock animals are characterised by several introgression events since domestication. In cattle, some of these events are known as secondary domestication, mostly due to the influence of wild aurochs males on domestic dam populations [54]. These ancient introgressions were combined with several long-distance migration events in the time before the industrial revolution and the subsequent foundation of modern breeds. Many local well-adapted strains have never been intensively managed and differentiated into regular breeds [3]. It is difficult (if at all necessary) to draw a clear line between the ancient and modern introgression events. Accordingly, the goal of this and the future studies aiming for restoration and conservation of the domestic breeds is not the ultimate recovery of the native genetic background but rather to stop ongoing erosion of genetic identity, to preserve diversity within the breed and, if possible, thereby to increase the proportion of native parts of chromosomes without the overall loss of diversity and increased rate of inbreeding.

## Conclusions

Each population is dynamic with a different identity and should be considered on an individual basis, but the following overall conclusions can be drawn from the results of this study. Combined phenotype, pedigree, and genome-wide information can be used to detect the “purest” individuals to be used as the nucleus for recovery of the native genetic background of the admixed domestic population. The ascertainment bias introduced in the construction of SNP arrays must be taken into consideration while estimating diversity and phylogeny parameters as well as for the removal of introgressed genetic material. The successful implementation of a conservation and recovery approach will benefit from a design which includes the most complete spectrum of possible source haplotypes.

## Supporting Information

S1 Fig The maximum likelihood (ML) tree inferred from genome-wide allele frequency data by methods implemented in the *TreeMix* program. A) The ML dendrogram of the relationships between 15 sampled populations rooted by African taurine N’Dama cattle. B) ML tree of 15 cattle populations assuming one migration event. C-E) Three variants of ML tree of 15 cattle populations assuming two migration events. F-G) Two variants of ML tree of 15 cattle populations assuming three migration events.
(PDF)

S2 Fig The *Admixture* analyses of two data sets at various levels of *K*, from 2 to 16. A dataset with all 76 Cika animals **(a)** and a reduced dataset with 26 Cika animals **(b)**. RHF, Red Holstein; FGV, Franken Gelbvieh; DFV, German Fleckvieh; MWF, Murnau—Werdenfelser; BBV, Braunvieh; OBV, Original Braunvieh; API, Pinzgauer; SIC, Cika; HRI, Istrian cattle; HRP, Slavonian—Syrmian Podolic; PMT, Piedmontese; RMG, Romagnola; HRB, Croatian Buša; IMB, Illyrian Mountain Buša; NDA, N’Dama.
(PDF)

S3 Fig Individual *D*_PS_ distance matrix in the two-dimensional space. Animals with *D*_*PS*_distance below global average were connected by thin grey line. A) Highlighting a set of 55 pure Cika animals recognised by merged information of haplotype, pedigree data and type traits classification, B) a set of 47 pure Cika animals recognised only by pedigree data were highlighted, C) a set of 58 pure Cika animals recognised only by type traits classification. RHF, Red Holstein; FGV, Franken Gelbvieh; DFV, German Fleckvieh; API, Pinzgauer; SIC, Cika; SIC-F, inbreed SIC animal; SIC*FGV*DFV, SIC related to FGV and DFV; SIC*API(I), SIC related to particular API animal; SIC*API(P), SIC related to API population; SIC-F*RHF, inbreed SIC animal related to RHF; SIC*RHF, SIC related to RHF.
(PDF)

S1 Table Correlations among type traits classifications, pedigree, genome-wide relationship (IBD) and the proportion of genes from *Admixture* analysis. (DOCX)
