# Supplementary figures and images for: Recovery of Native Genetic Background in Admixed Populations Using Haplotypes, Phenotypes, and Pedigree Information – Using Cika Cattle as a Case Breed
Source: PLoS One. 2015 Apr 29;10(4):e0123253. doi: 10.1371/journal.pone.0123253 (PMC4414476; doi:10.1371/journal.pone.0123253)

A)

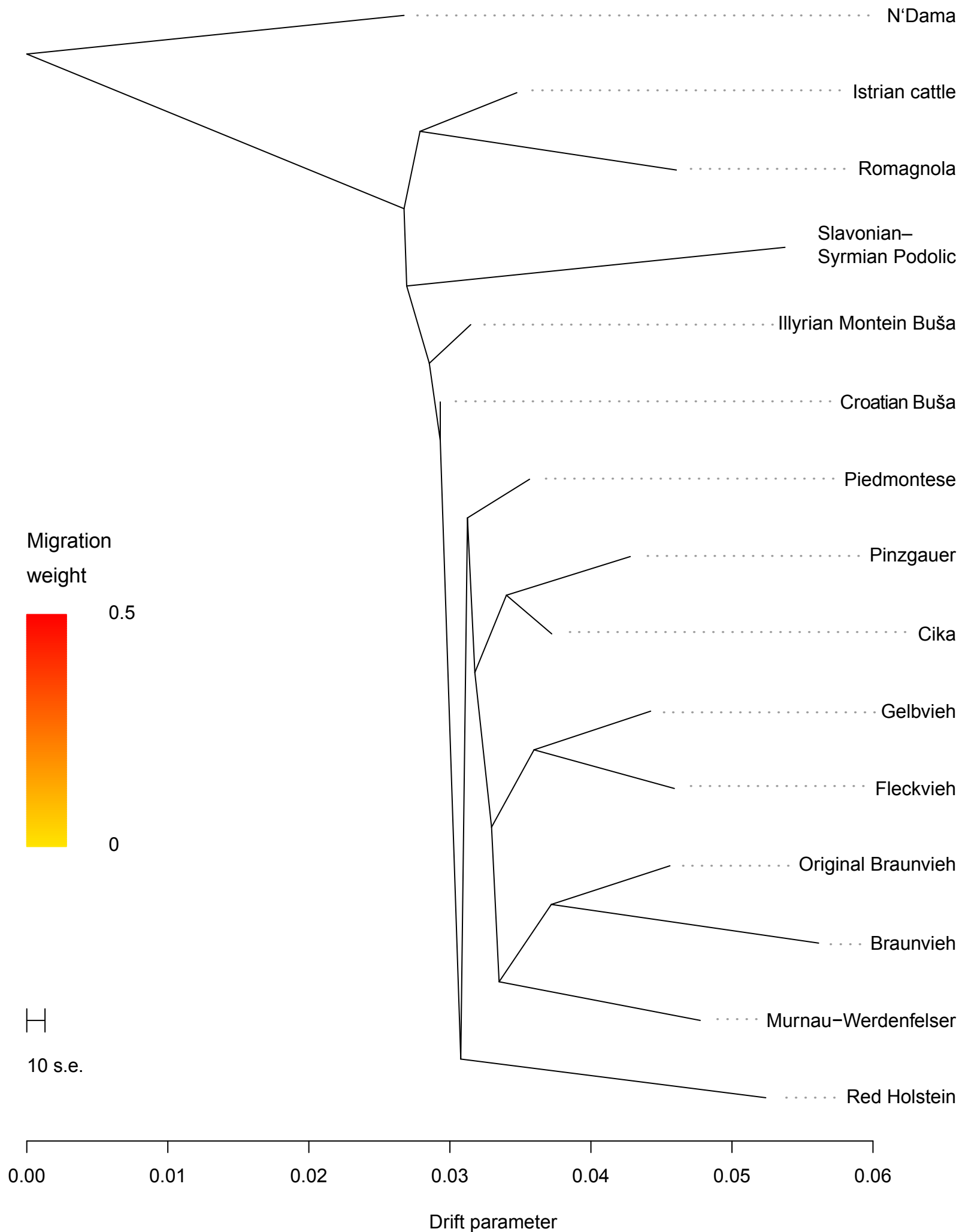

B)

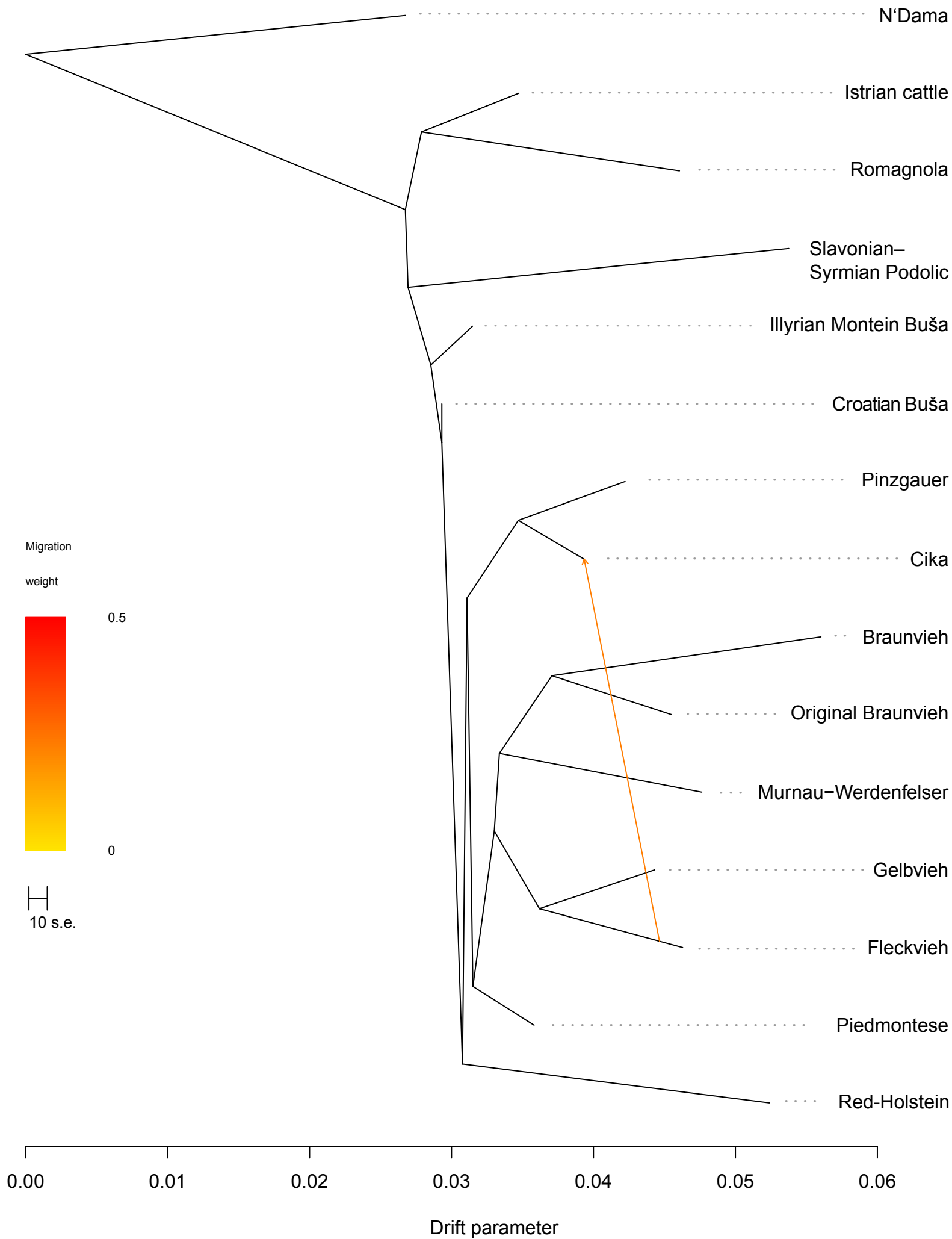

C) ones

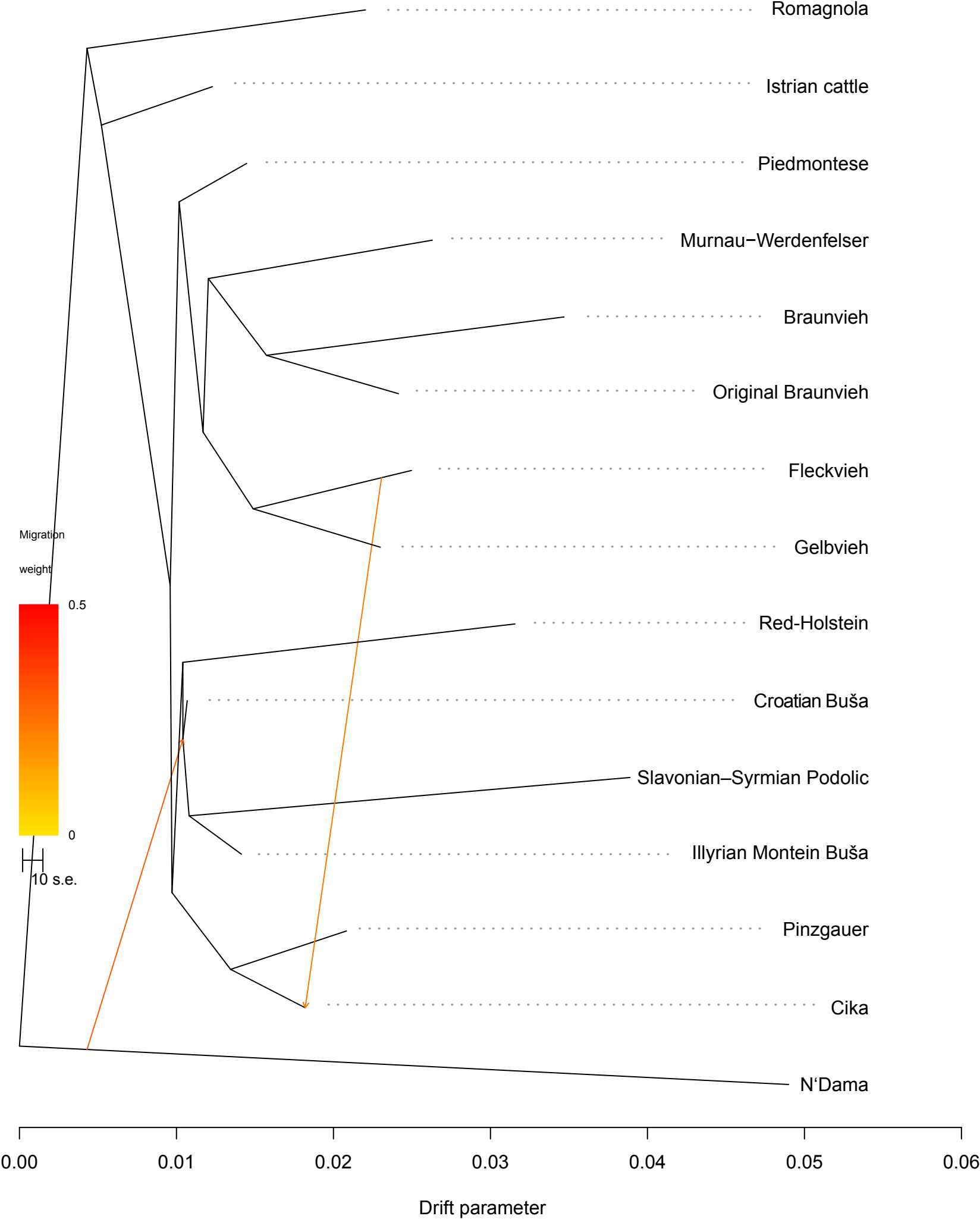

D) ones

Migration weight

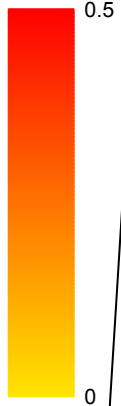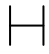

10 s.e.

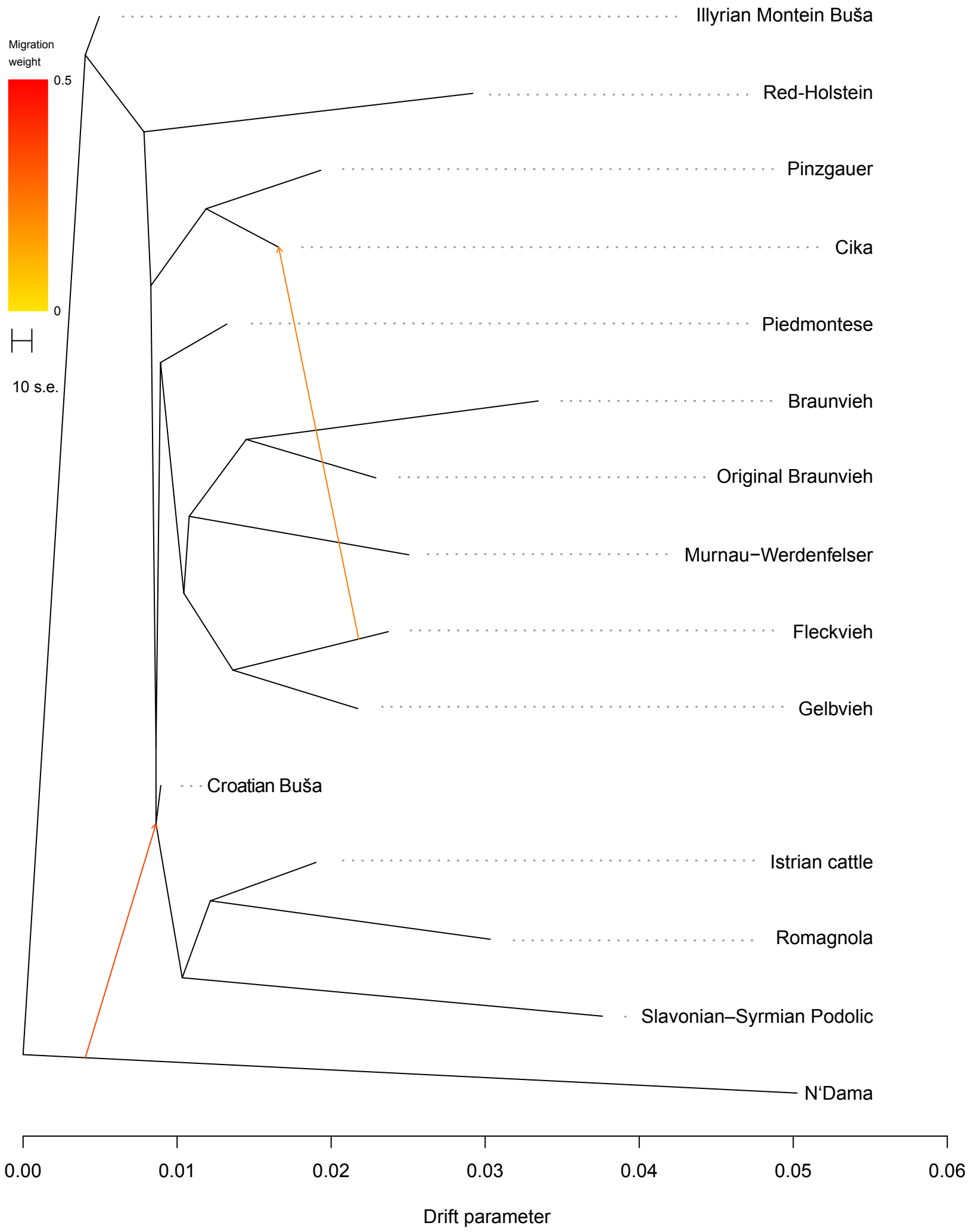

E) three-times

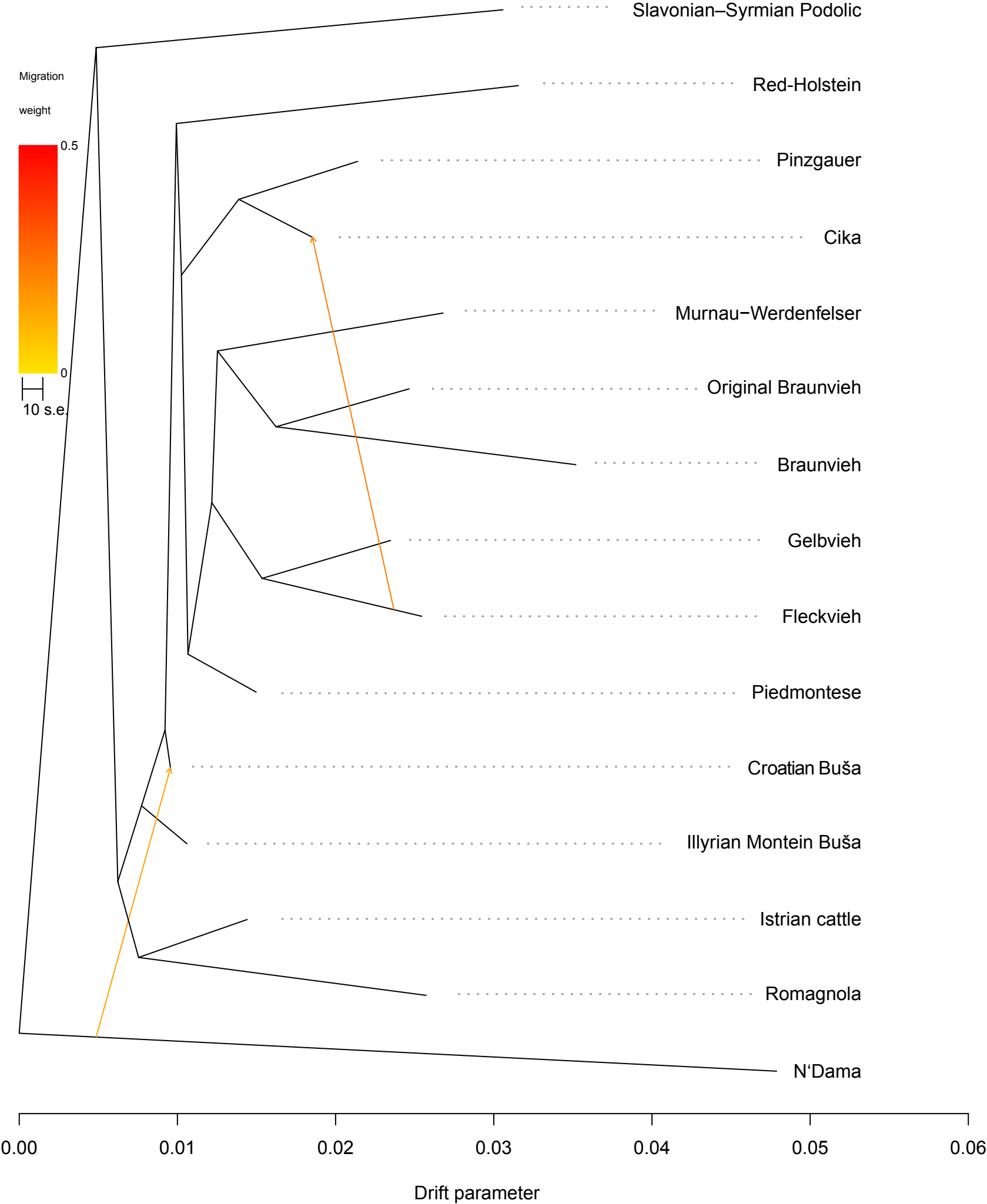

F) ones

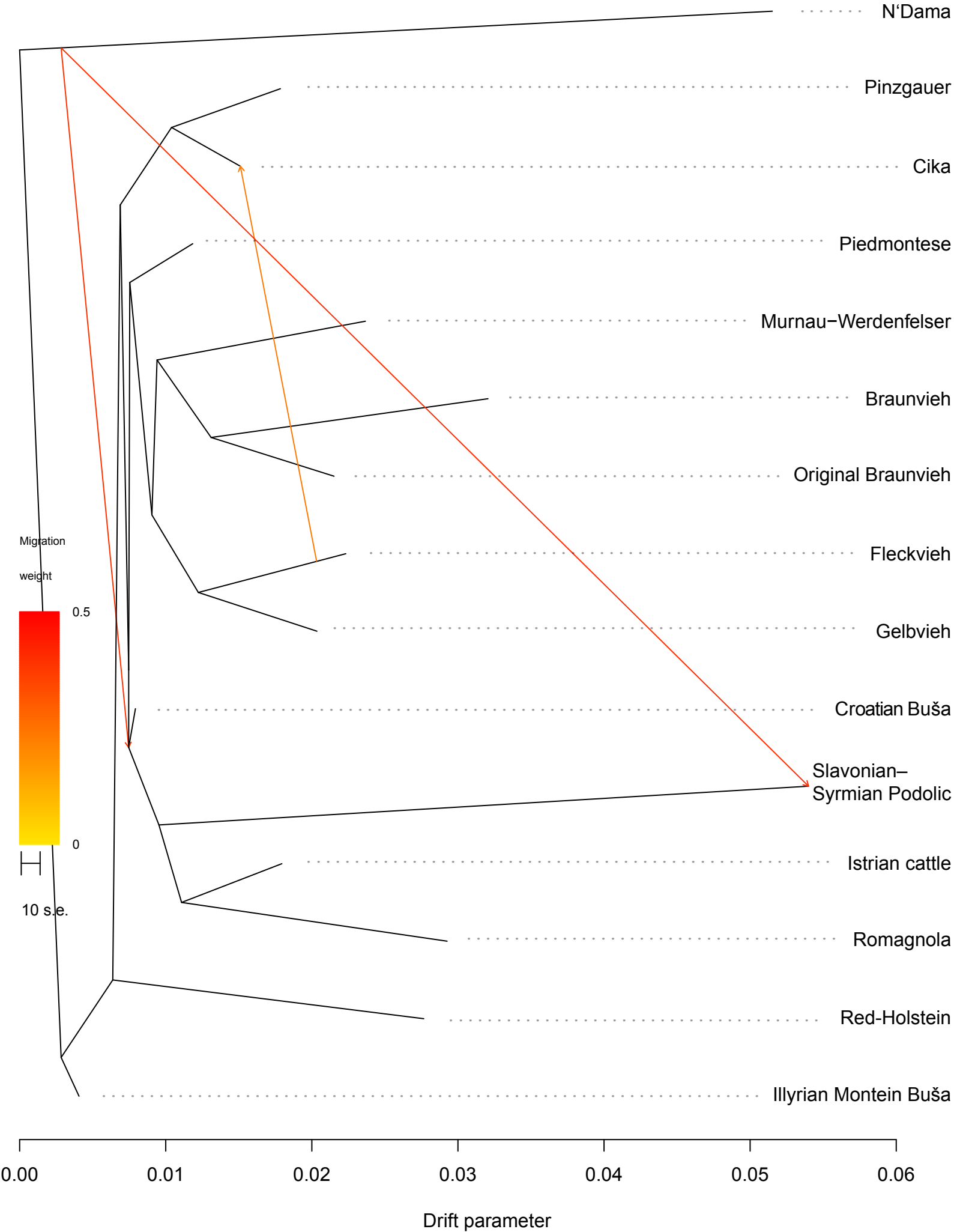

G) four-times

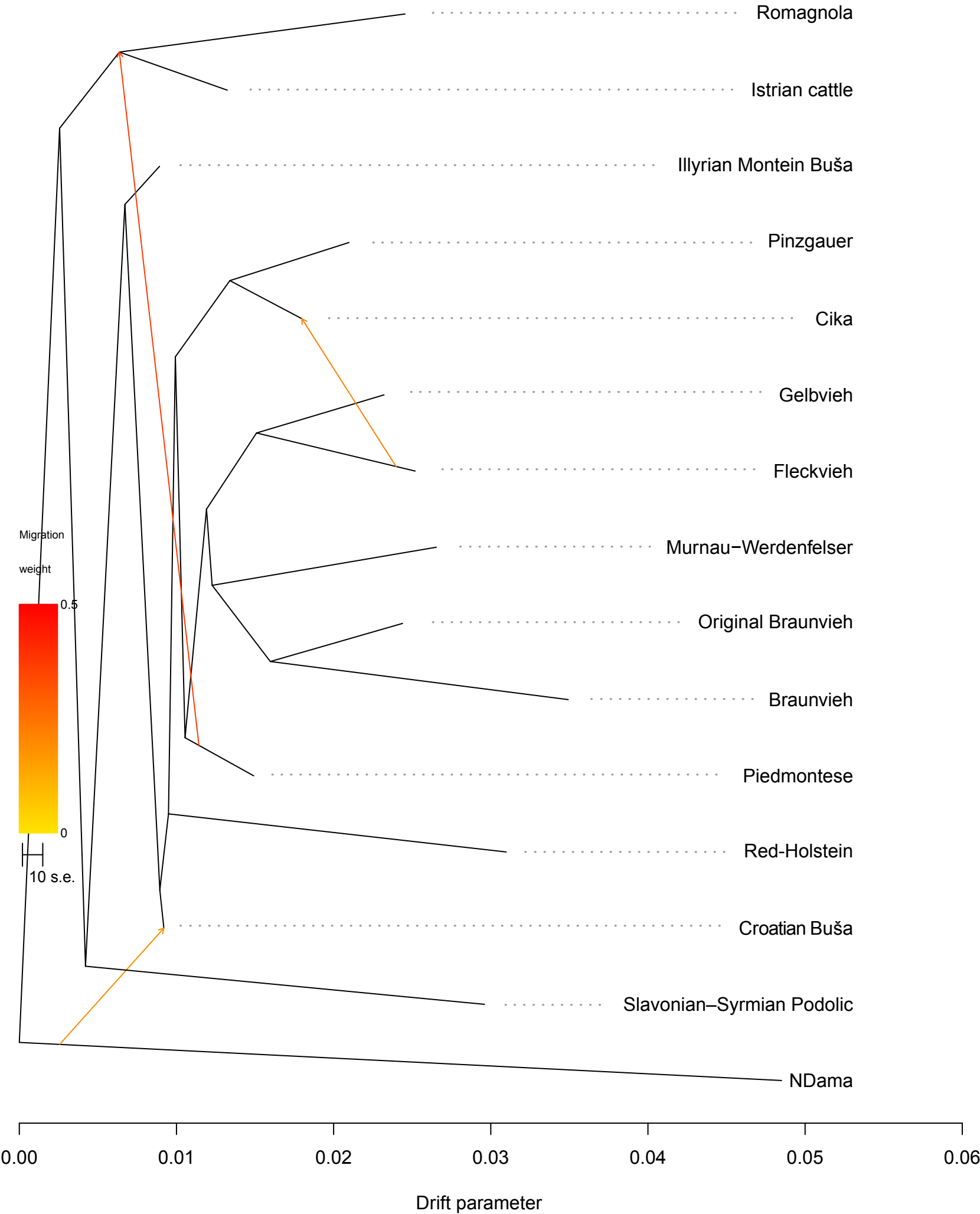

Supplement: S1 Fig — A) The ML dendrogram of the relationships between 15 sampled populations rooted by African taurine N’Dama cattle. B) ML tree of 15 cattle populations assuming one migration event. C-E) Three variants of ML tree of 15 cattle populations assuming two migration events. F-G) Two variants of ML tree of 15 cattle populations assuming three migration events. (PDF) [file pone.0123253.s001.pdf]

A)

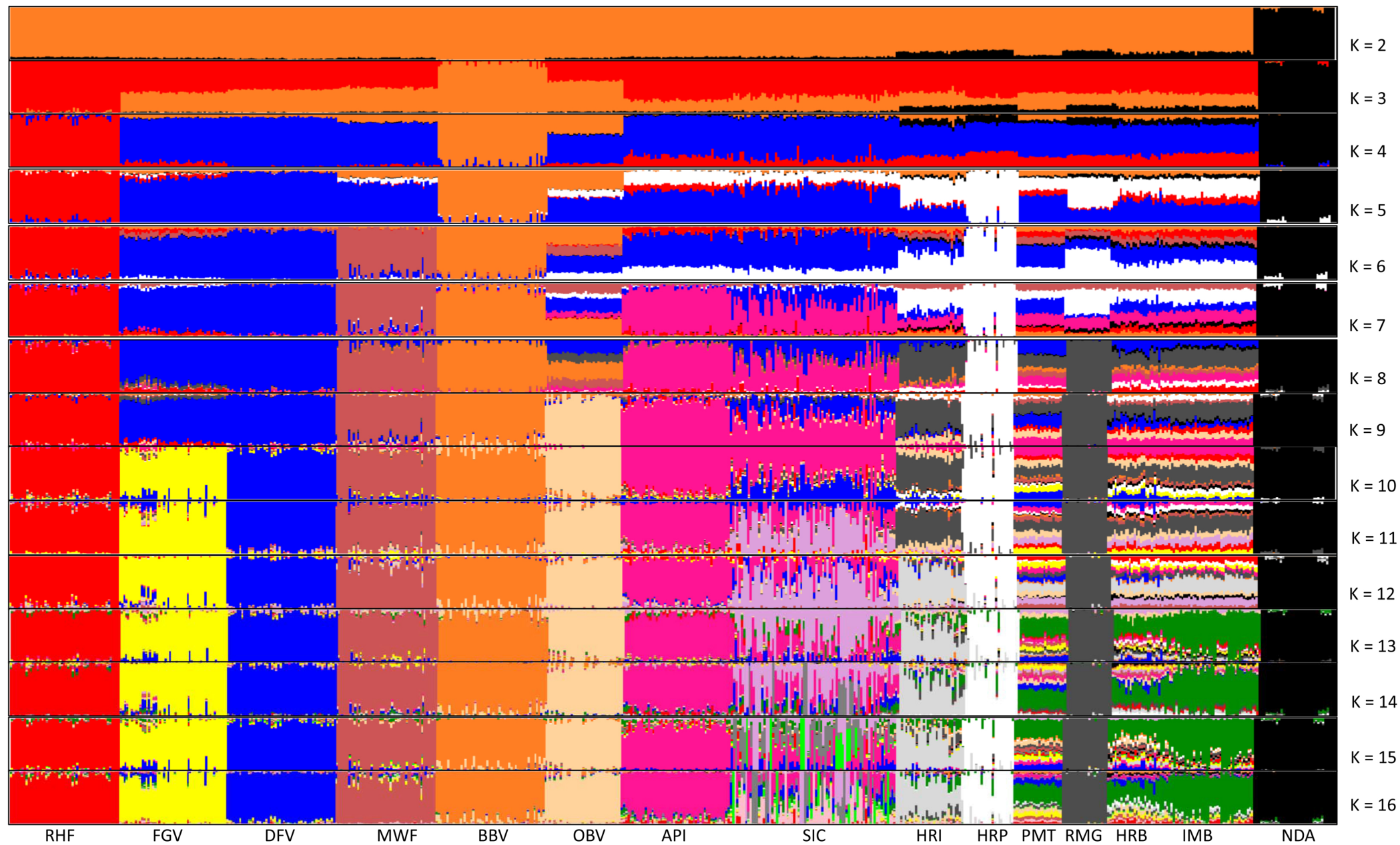

B)

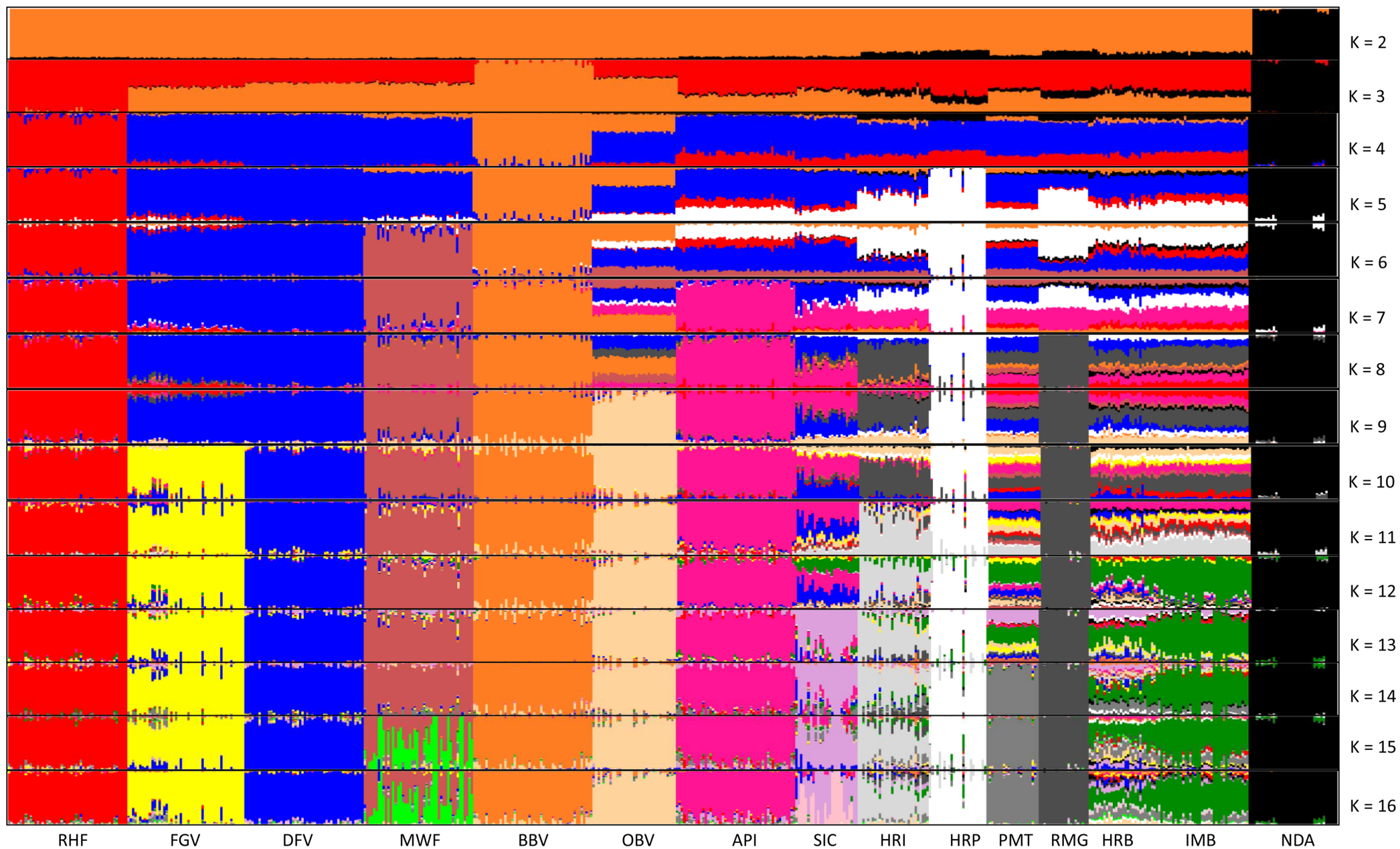

Supplement: S2 Fig — A dataset with all 76 Cika animals (a) and a reduced dataset with 26 Cika animals (b). RHF, Red Holstein; FGV, Franken Gelbvieh; DFV, German Fleckvieh; MWF, Murnau—Werdenfelser; BBV, Braunvieh; OBV, Original Braunvieh; API, Pinzgauer; SIC, Cika; HRI, Istrian cattle; HRP, Slavonian—Syrmian Podolic; PMT, Piedmontese; RMG, Romagnola; HRB, Croatian Buša; IMB, Illyrian Mountain Buša; NDA, N’Dama. (PDF) [file pone.0123253.s002.pdf]

A)

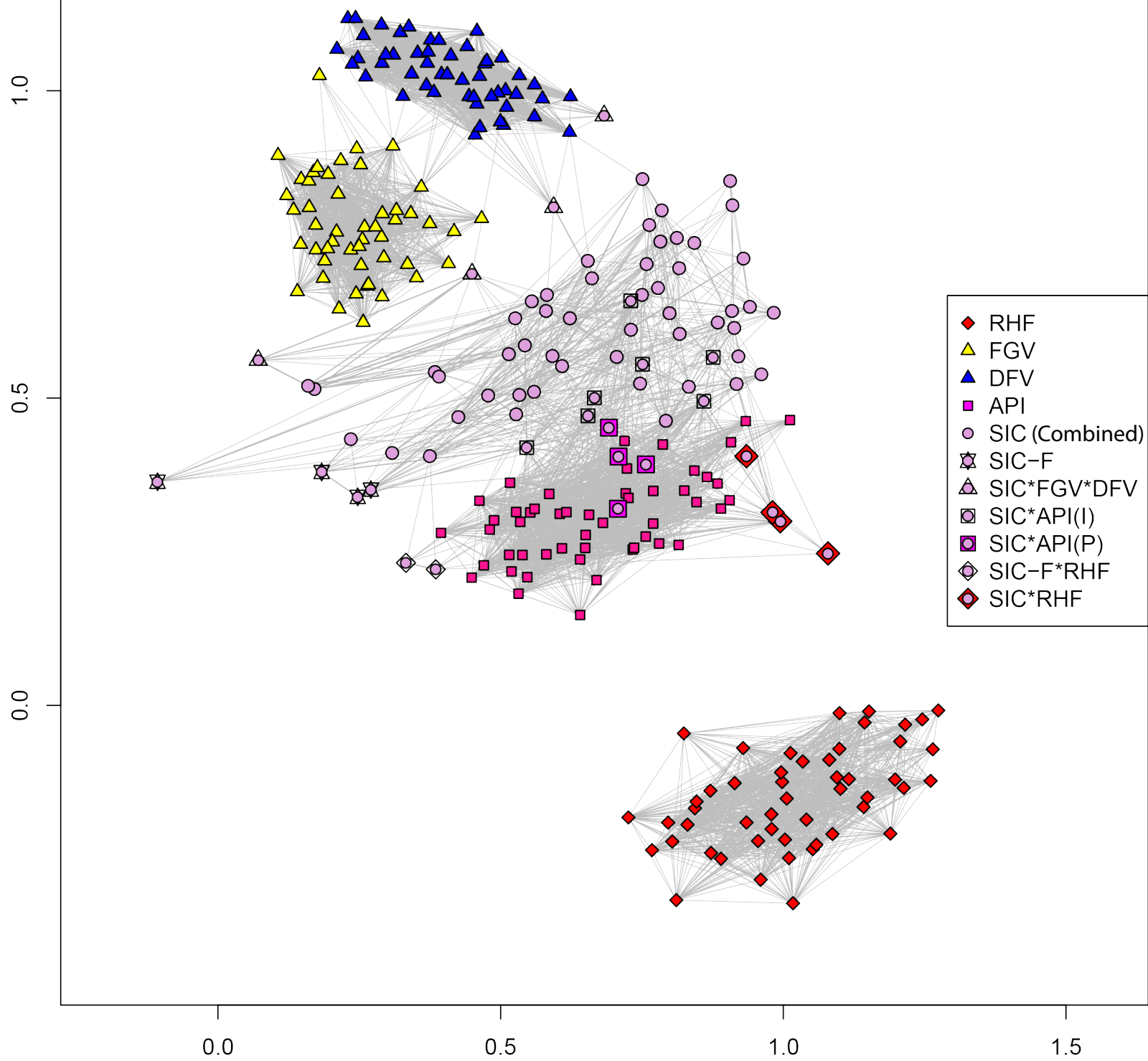

B)

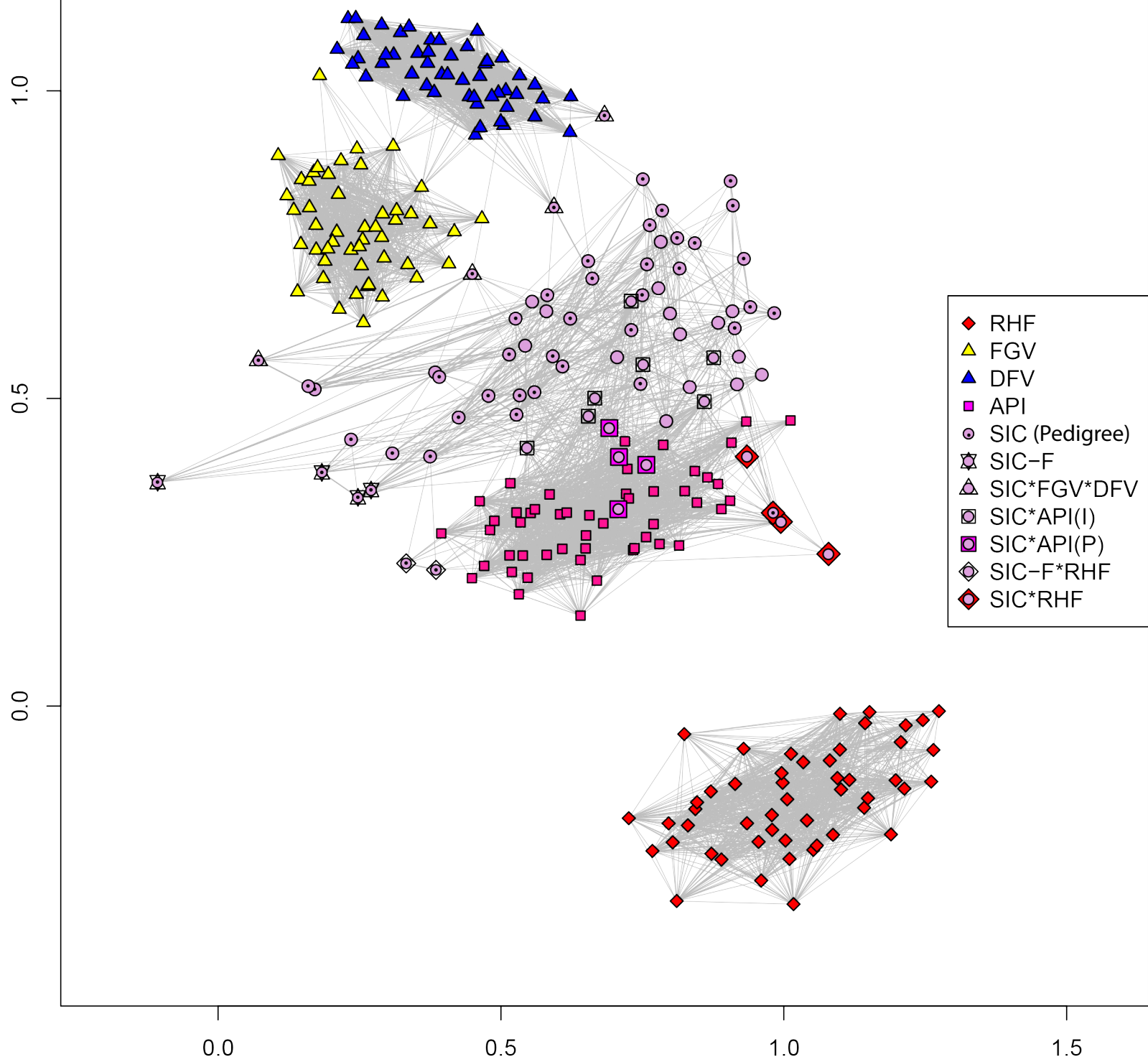

C)

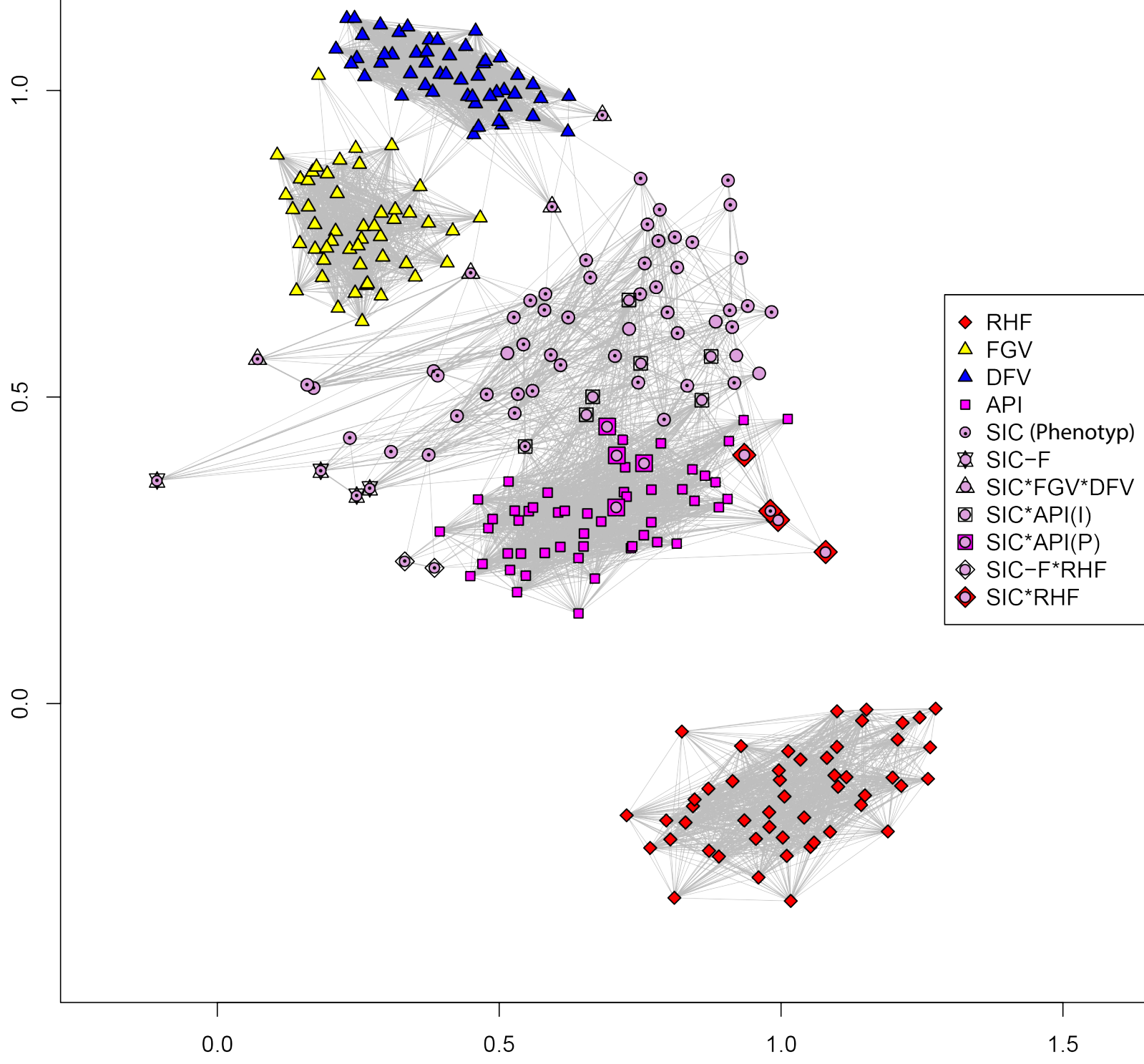

Supplement: S3 Fig — Animals with D PSdistance below global average were connected by thin grey line. A) Highlighting a set of 55 pure Cika animals recognised by merged information of haplotype, pedigree data and type traits classification, B) a set of 47 pure Cika animals recognised only by pedigree data were highlighted, C) a set of 58 pure Cika animals recognised only by type traits classification. RHF, Red Holstein; FGV, Franken Gelbvieh; DFV, German Fleckvieh; API, Pinzgauer; SIC, Cika; SIC-F, inbreed SIC animal; SIC*FGV*DFV, SIC related to FGV and DFV; SIC*API(I), SIC related to particular API animal; SIC*API(P), SIC related to API population; SIC-F*RHF, inbreed SIC animal related to RHF; SIC*RHF, SIC related to RHF. (PDF) [file pone.0123253.s003.pdf]
