# Supplementary material for: Recovery of Native Genetic Background in Admixed Populations Using Haplotypes, Phenotypes, and Pedigree Information – Using Cika Cattle as a Case Breed
Source: PLoS One. 2015 Apr 29;10(4):e0123253. doi: 10.1371/journal.pone.0123253 (PMC4414476; doi:10.1371/journal.pone.0123253)
Supplement: S1 Table — (DOCX) [file pone.0123253.s004.docx]

**Supplementary table S1:** Correlations among type traits classifications, pedigree, genome-wide relationship (IBD) and proportion of genes from *ADMIXTURE* analysis.

|  | **Type traits classification** | **IBD (SIC-API)** | **SIC genes (Pedigree)** | **API genes (Pedigree)** | **SIC genes (*ADMIXTURE*)** | **API genes (*ADMIXTURE*)** |
| --- | --- | --- | --- | --- | --- | --- |
| **Type traits classification** | 1.00 |  |  |  |  |  |
| **IBD (SIC-API)** | 0.65*** | 1.00 |  |  |  |  |
| **SIC genes (Pedigree)** | -0.73*** | -0.67*** | 1.00 |  |  |  |
| **API genes (Pedigree)** | 0.71*** | 0.69*** | -0.98*** | 1.00 |  |  |
| **SIC genes (*ADMIXTURE*)** | - 0.53*** | -0.55*** | 0.60*** | -0.58*** | 1.00 |  |
| **API genes (*ADMIXTURE*)** | 0.65*** | 0.95*** | -0.70*** | 0.72*** | -0.73*** | 1.00 |

SIC, Cika; API, Pinzgauer
